# Advances in biomonitoring technologies for women’s health

**DOI:** 10.1038/s41467-025-63501-3

**Published:** 2025-09-26

**Authors:** Shaghayegh Moghimikandelousi, Lubna Najm, Yerim Lee, Fereshteh Bayat, Akansha Prasad, Shadman Khan, Aishwarya Bhavan, Wei Gao, Zeinab Hosseinidoust, Tohid F. Didar

**Affiliations:** 1 School of Biomedical Engineering, McMaster University, Hamilton, ON Canada; 2 Department of Mechanical Engineering, McMaster University, Hamilton, ON Canada; 3 Andrew and Peggy Cherng Department of Medical Engineering, Division of Engineering and Applied Science, California Institute of Technology, Pasadena, CA USA; 4 Department of Biochemistry and Biomedical Sciences, McMaster University, Hamilton, ON Canada; 5 Department of Chemical Engineering, McMaster University, Hamilton, ON Canada; 6 Farncombe Family Digestive Health Research Institute, McMaster University, Hamilton, ON Canada; 7 Michael DeGroote Institute for Infectious Disease Research, McMaster University, Hamilton, ON Canada

**Keywords:** Biomedical engineering, Health care, Biomaterials, Materials for devices

## Abstract

In global healthcare systems, sex and gender biases have favored cisgender males, which has led women and transgender individuals to be understudied and underrepresented in medical literature. Thus, these populations are largely overlooked in health policy making. Persistent gender inequalities, socioeconomic divides, and racial-ethnic discrimination, particularly in low-resource communities, have exacerbated women’s health concerns, delaying advancements in care and accessibility. However, recent years have seen the emergence of tracking technologies and wearable devices that enable long-term biomonitoring of key health biomarkers which promise to facilitate early disease diagnosis for women from all walks of life. These innovations value education and accessibility, which can break down barriers to health care access and management that has affected generations of women around the world. This review discusses emerging biomonitoring technologies for diagnosing and managing critical women’s health conditions as defined by the World Health Organization, including breast and gynecological cancers, vaginal infections, fertility, pregnancy and post-menopausal osteoporosis. Additionally, we examine the current commercial landscape of women’s health technologies, highlighting barriers to adoption, such as medical insurance access and socioeconomic status, as well as discuss opportunities for future innovation.

## Abstract (web summary)

In global healthcare systems, sex and gender biases have led to a health gap that affects diverse populations of women. This review highlights emerging wearable, biomonitoring, and diagnostic technologies to address this health gap, examines the commercial landscape and barriers to adoption, and discusses opportunities for future innovation.

## Introduction

Despite many achievements in women’s civil rights over the past century, gaps in healthcare, medical research, sociocultural standing and economic power continue to affect women’s lives in the modern day^1,2^.

From an economic perspective, women have struggled with attaining equal resources to men because of barriers in accessing education, job opportunities, safe housing, and healthcare services^1^. For instance, women’s opportunities in receiving medical insurance coverage and healthcare infrastructure resources are limited and often do not fully encompass women’s health needs^3^. This leads to nearly 10.8 million women remaining medically uninsured in the USA, and 1.5 billion women and girls worldwide lacking access to healthcare facilities or resources^3,4^. The healthcare gap is exacerbated amongst populations of women in middle to low-income communities and transgender people^5,6^. In addition, funding and investment for health research and medical innovation has a biological sex bias^7^. Despite $198 billion USD being invested in health research in the USA, only 4% is allocated to women’s cancer and 1% for all other female conditions^7^. Additionally, conditions such as hepatitis B, which affects men 2$$\times$$ more than women, receive 41$$\times$$ more attention and funding in clinical or health research compared to conditions predominantly affecting women^7^. This disparity stems from the viewpoint that ‘women’s health’ is niche, resulting in women-prevalent conditions being neglected and understudied by funding agencies, policy makers and researchers^7^.

Other than research funding, biological sex biases affect clinical and medical studies^8^. Historically, biological male participants, animal models and cell lines have been the bases of medical studies, which become generalized to women. These studies do not consider biological sex differences present in genetics, hormones, symptomology and bodily functions^8^. This bias results from the fear of negatively impacting women’s fertility and pregnancy, resulting in only 37% of women representation amongst academic journals^8^. Furthermore, amongst in vitro cell studies, 70% of cell lines used are derived from biological males^8^. Consequently, the lack of sex considerations and limited health research is highly associated with increased incidence of harmful, ineffective or inappropriate treatment strategies implemented for females^8^.

The presence of these gaps leads to women often feeling limited in their understanding of their own bodies and unaware of their health choices^2^. Personalized, cost-effective and reliable technologies can address the concerns that women face regarding the overall limited medical data on women-prevalent conditions^7^. Women themselves have also shown interest in adopting wearable and wireless technologies for tracking their own health. For instance, there is a 77% acceptability rate towards using ovulation tracking via bracelet wearables^9^. Transgender individuals also express interest in leveraging technologies for understanding their health. In qualitative studies conducted amongst transgender individuals, key factors listed for these technologies include the promotion of fluidity and gender identity, ability to support different kinds of transitions, and easy-to-manage user interfaces for data concerning transition and gender identity^6,10^.

**Fig. 1:**
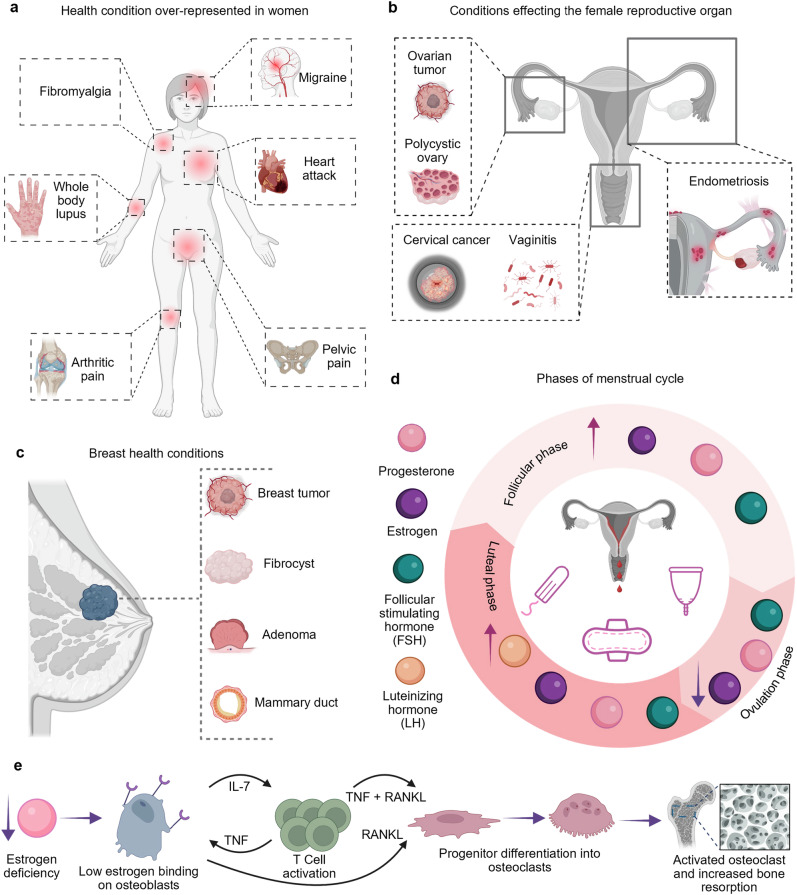
Overview of women’s health conditions. **a** Depiction of conditions over-represented in women. **b** Schematics are shown for the progression of gynecological conditions. **c** Depiction of breast health conditions, including breast tumors, adenomas, mammary duct abnormalities, and fibrocystic tissues. **d** Illustration of the monthly female hormonal cycle, highlighting the different phases of menstrual cycle. **e** The role of female hormones in the progression of postmenopausal osteoporosis and the activity of bone-related cells. Created in BioRender. Hosseinidoust, Z. (2025) https://BioRender.com/t20f565.

The World Health Organization (WHO) and various government agencies have reported that the most prevalent diseases and concerns amongst women are cancer, reproductive health, maternal health, and osteoporosis (Fig. 1a–e)^11,12^. Moreover, biomonitoring technologies, such as wearable devices and point-of-care (POC) diagnostic tools, have gained attention due their ability to enable early detection and long-term health management of these conditions. This is done by measuring and analyzing personalized vitals, hormones and biomarkers^5^. In this review, we discuss the emerging and accessible technologies for the diagnosis and monitoring of women’s health amongst diverse populations, based on the WHO’s reports. We describe the advantages and limitations of these emerging technologies, identifying gaps and areas in need of more attention or development. Furthermore, we highlight how artificial intelligence (AI) and machine learning (ML) algorithms present an unparalleled opportunity for improving women’s health diagnostics by enhancing the accuracy of current tools in predicting health problems^13^.

## Wearable technologies for biomonitoring

**Fig. 2:**
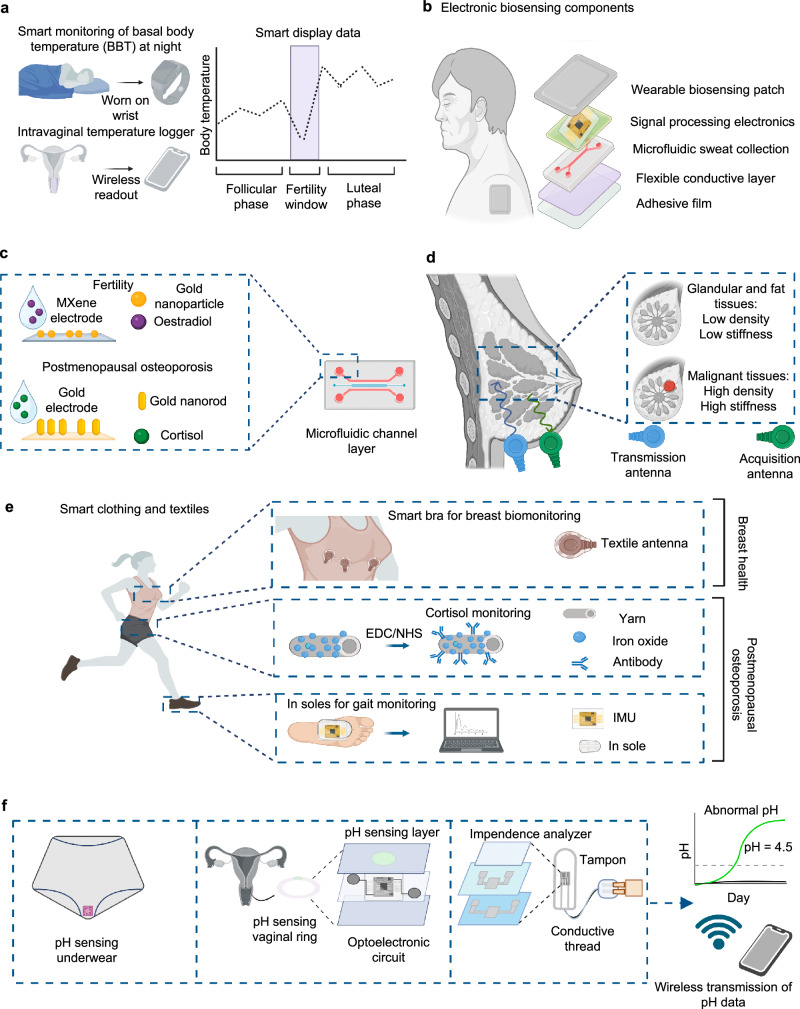
Depiction of wearable biomonitoring technologies. **a** Nocturnal BBT monitoring with wrist wearable and intravaginal temperature monitoring with wireless logger. **b** Components of adhesive patches in hormonal monitoring. **c** Adhesive patches in microfluidics for oestradiol and cortisol monitoring. **d** Antenna pair system in healthy breast tissues compared to malignant tumor tissue. **e** Smart clothing and textiles, such as smart bras for breast cancer monitoring, conductive yarn for sweat cortisol monitoring and in-sole sensors for osteoporosis gait monitoring. **f** Wireless biosensor using pH sensitive chips embedded in underwear, vaginal rings and tampon surfaces. Created in BioRender. Hosseinidoust, Z. (2025) https://BioRender.com/t20f565.

Advancements in bioengineered wearable technologies have enabled real-time, long-term self-monitoring, providing women with reliable health data and gaining traction in both research and clinical practice (Fig. 2)^14^. This section focuses on wearable technologies for monitoring female prevalent conditions recommended by the WHO (Supplementary Table 1)^11,12^. Additionally, critical conditions that remain underrepresented in wearable health monitoring solutions are discussed, such as cardiovascular disease (CVD) and chronic pain, because of the limited experimental data available on these conditions to inform wearable technology design parameters for women^15,16^. Literature on these conditions mainly report observational studies and qualitative assessments from small pools of women^16^.

### Biometric monitoring for fertility and pregnancy health

Monitoring fertility and maternal health is crucial for both the mother and child, as 810 and 6700 maternal and fetal deaths, respectively, occur every day^17,18^. Additionally, the WHO reports that, annually, 40 million women develop long-term health conditions during pregnancy^19^. If left improperly diagnosed or unaddressed, these conditions negatively impact women’s lives for years after giving birth, showing a need for health monitoring solutions^19,20^. Women in low-income and low-middle income countries can especially benefit, as 92% of maternal deaths in these countries are preventable by addressing the lack of access to high-quality healthcare resources, well-informed clinical guidelines and reliable data on women’s reproductive health^20^.

Biometric parameter changes, trackable with wearable devices, are characterized by physiological fluctuations throughout the menstrual cycle and pregnancy^21^. These parameters include basal body temperature (BBT)^22^, peripheral temperature on the skin^23^, sweat rate^24^, and heart rate^25^. Some wearables offer a non-invasive alternative for measuring these parameters compared to traditional clinical assessments that require physician facilitation. These include temperature measurements recorded with a thermometer either orally, rectally or intravaginally^26–28^, thermocouple or infrared sensor heat mapping conducted in clinics^23^, and off-body sweat sample analyses performed in laboratories^29^.

Wearable devices for BBT monitoring should be capable of detecting fluctuations in the range of 0.5–0.8 °C, as this is the typical variation observed throughout the menstrual cycle^30,31^. Generally, estradiol levels increase throughout the follicular phase, peaking at ovulation, which is associated with increased vasodilation and body cooling^32^. Subsequently, after ovulation, high progesterone levels lead to body heating by affecting the hypothalamus and central nervous system to increase the body’s thermoregulatory setpoint^27,33^. The most common BBT monitoring wearables are wrist-worn activity trackers. However, the challenges with measuring daily BBT through smart activity watches and bracelets are that fluctuations throughout the day, depending on level of activity, lead to fluctuating accuracy^31^. These trackers are best suited for detecting biometric changes at night, when the body is at rest^31^.

BBT monitoring via wearable technologies for intravaginal temperature tracking offers a stable and accurate method for continuous reproductive health assessment, compared to manual or wrist-worn recorded temperature measurements at specific timepoints each day (Fig. 2a)^34^. Intravaginal loggers, once inserted, provide automated real-time temperature measurements over multiple days without the need for removal^35^. These devices, such as OvuSense, show 69.2% acceptability rate amongst women, with 76.9% reporting convenience and comfort during use over three menstrual cycles^36^.

Wearable devices that monitor biometrics specific to pregnancy can provide an overall surveillance of the pregnancy’s trajectory^37^. Pregnancy-specific biometrics include maternal heart rate (interchangeable with pulse rate but focused on mother rather than fetus)^38^, maternal cardiac function (traditionally monitored via electrocardiogram (ECG) or transthoracic echocardiogram (TTE))^39^, uterine contractions^40^, blood oxygenation^39^ and sleep patterns^41^. For instance, wearable soft sensor systems leveraging traditional methods, such as ECG, electrohysterogram (EHG), and Doppler ultrasound, that can be worn over the abdomen provide a comprehensive solution for maternal biometrics tracking^42^. Moreover, sensors integrated into conductive clothing, referred to as smart textiles, offer a non-invasive approach to monitoring uterine contractions, maternal heart rate, and respiration^43,44^. Although still in early development, large-scale clinical testing is required to refine smart textiles for broader adoption.

Gestational diabetes mellitus (GDM) is a common complication developed during pregnancy that can benefit from biometric-based wearable technologies. GDM affects ~14% of pregnant women worldwide and leads to 10$\times$ increase in the risk of postpartum type 2 diabetes^45–48^. GDM is characterized by an intolerance towards glucose that develops during the second or third trimester of pregnancy, with lifestyle being the number one risk factor^45,49^. Lifestyle factors impacting the likelihood of developing GDM include activity levels, body mass index (BMI), stress levels, nutrition, and sleep patterns^45,50,51^. Wearable technologies are designed to continuously track and record pregnancy and diabetes biometric parameters, such as physical activity indicators (e.g., number of steps, sitting and standing occurrences, actigraphy, heart rate), blood and interstitial glucose levels, renal function, and circadian rhythm^41,45,50–53^.

Commercial wearable tracking devices worn on the hips and wrists capture specific biometric data over long time frames, sufficient for second and third trimester monitoring and GDM detection^45^. Such devices include the Abbott FreeStyle Libre Pro^45^ (interstitial glucose levels), Medtronic Guardian Connect^50^ (tissue glucose under the skin), CamNTech ActiHeart device^45^ and Firstbeat Bodyguard 2^50^ (focused on heart rate variability to detect abnormalities), GENEActiv^45^ (focused on sleep analysis), Exsed from UKK-Institute^50^ (focused on tracking movement), Vivosmart 3^50^ and Fitbit Charge 3^53^ (step count and speed). Combining these data onto one wearable device can provide a comprehensive lifestyle profile for pregnant women. For instance, Ava Bracelet trackers capture data on movement during sleep, sleep duration, pulse rate, breathing rate and skin peripheral temperature^41^. These biometrics, collectively, provide results on sleep analysis, emotional states and stress levels throughout pregnancy and postpartum^41^.

On the research front, miniaturized electrochemical biosensors or immunosensors targeting biomarkers associated with disease progression show promise for GDM monitoring^50,54^. For instance, an electrochemical wearable technology, combining miniaturized circuitry, cost-effective material and microneedle sample collection, has been proposed for detecting β-hydroxybutyrate and/or lactate alongside glucose. The former two being key components for the diabetes ketosis pathway seen in GDM^55,56^. This technology achieved a LOD of 50 μM with 95% stability over 6 h in artificial interstitial fluid^56^. Cystatin C (Cys-C) is another such biomarker shown to increase severity in the presence of GDM. Cys-C is associated with multiple metabolic and physiological mechanisms as well as adverse immunological, renal and fetal growth effects^52^. In a label-free miniaturized electrochemical immunosensor wearable, leveraging screen-printed electrodes coated with MXene and gold nanoparticles, Cys-C ranges of 50–5000 ng/mL were monitored over 30 days when placed on skin^52,57^. Multiplex detection with dopamine and uric acid was also used to provide comprehensive insight into the renal function during GDM^57^.

Another important aspect of GDM management is reducing reported feelings of failure, anxiety, and powerlessness many pregnant women face after receiving a diagnosis of GDM^50^. By tracking both self-reported and wearable monitored biometrics on accessible mobile health (mHealth) applications, pregnant women can remain informed about their own health and lifestyle decisions^46,58^. mHealth applications tracking nutritional health and blood glucose levels, inputted manually 3–6 times a day, are amongst the most used worldwide, such as GDm-Health (UK), Dnurse (China), MoTHER (Australia), MyDiabby (France), Diamond (Australia), and Pregnant+ (Norway)^58^.

Despite the notable advancements on this front, the main barrier faced is the need for accurate data interpretation to direct proper management of GDM^49^. Currently, even with proper utilization of wearables and smartphone applications, many women seek healthcare provider support to understand the significance of collected mHealth data. These women report hesitance in making health decisions or behavioral changes without professional feedback^50^. As such, wearable and mHealth technology are implemented as an enhancement to the clinical care for pregnant women rather than as a complete substitute^46^.

Next generation designs of these mHealth technologies are expected to leverage remote professional feedback tools built into user interfaces, which can increase adoption amongst different demographics^50,51,58^. One example of such emerging wearable devices includes the Fitbit Flex activity and nutrition tracker, which incorporated a feedback and encouragement text messaging system to promote positive behaviors and lifestyle change goals^48^. Another step for wearable pregnancy monitoring involves launching community outreach and education initiatives focused on the wearables technologies in clinics and healthcare systems, amongst pregnant women and their healthcare providers. Such initiatives will bridge digital literacy gaps and encourage users to adopt these technologies^59^. Wireless and remote communication is also key, as it enables remote monitoring between pregnant women and their clinicians, allowing for telemedicine application in pregnancy care^60^. Telemedicine has the advantage of allowing pregnant mothers to monitor their health alongside a health professional from the comfort of their home, reducing need for clinic visits^60^. However, for these initiatives and communication platforms, diverse populations of women need to be considered, including those from different cultural backgrounds, age groups, socioeconomic standings and those with limited internet access^61,62^.

### Hormonal monitoring wearables

While general biometric wearables, such as smartwatches and fitness trackers, provide useful data, they fail to capture the complexity of menstrual cycles, hormone therapies, and their physiological implications. Integrating hormone biosensors into existing platforms could enhance diagnostic capabilities and lead to more personalized healthcare solutions.

Hormonal fluctuations play a central role in the menstrual cycle, influencing the fertility window and ovulation^63^. In addition to progesterone and estradiol, other key hormones of the menstrual cycle include follicle-stimulating hormone (FSH), estrone-3-glucuronide (E1G), cortisol and pregnanediol glucuronide (PdG), which dip and peak at different phase of menstrual cycle^64^. For example, sweat-based nanobiosensors integrated into microfluidic wearable devices show promise in detecting hormones non-invasively (Fig. 2b, c)^63,65^. Aptamers functionalized onto gold nanoparticle-MXene (AuNP-MXene) electrodes enable highly sensitive hormone detection at picomolar concentrations, significantly enhancing predictive accuracy^66^.

Cortisol is also a key biomarker of reduced bone health as it influences calcium absorption and bone metabolism leading to osteoporosis^67,68^. Electrochemical detection of osteoporosis using sweat-based wearables, including microfluidics, microneedles, and textiles, enable non-invasive cortisol detection (Fig. 2b,c)^66,69,70^. Smart textiles incorporating modified carbon yarn fibers coated with iron oxide (Fe₂O₃)-based conductive nanomaterials can selectively detect cortisol via chemically crosslinked monoclonal antibodies in a miniaturized nanobiosensor platform (Fig. 2e)^69,71^. In addition, during pregnancy, cortisol monitoring is essential, as cortisol levels are 2–4$\times$ higher when pregnant *vs*. non-pregnant, which has been correlated with bone loss^72,73^. One device that has the potential to address this risk by targeting cortisol monitoring in pregnant women is U-RHYTHM, which can monitor 24-hr ultradian rhythm fluctuations of cortisol and its derivates in the serum of pregnant women with different body weights^25,74^. By collecting data on multiple hormonal signals, devices such as U-RHYTHM enable deeper investigations of underlying biological mechanisms associated with changes in these signals^25,74^.

Despite established correlations between hormonal changes and women-prevalent conditions, for chronic pain^75^, endometriosis^76^, polycystic ovarian syndrome (PCOS)^77^, osteoarthritis^78^, CVD^79^, and lung cancer^80^, no wearable technologies currently exist target these conditions^81^. For instance, endometriosis, characterized by chronic menstrual pain, is associated with abnormal hormone levels^76^. Gonadotropin-releasing hormone (GnRH) agonists, commonly used for treatment, ultimately suppress FSH, LH, and estrogen levels, leading to temporary menopause-like states and reducing bone density^82^. However, no wearable devices exist for real-time hormone tracking before, during, or after endometriosis treatment. Similarly, testosterone is the key biomarker for PCOS diagnosis, yet there are no at-home biomonitoring solutions for its detection^83^. Estrogen is also particularly relevant for various health conditions, including migraines, fibromyalgia, CVD, and lung cancer^80,84–86^.

#### Transgender considerations in wearables design

The literature on women’s health wearable technologies focuses disproportionately on design parameters, usability and experiences of cisgender women. However, a gender identity gap is also present in medical practice^87^. Little consideration in medical literature has been given to transgender experiences, before, during and after undergoing transition^88^. Fertility is another consideration regarding this health gap, as 76% of transgender individuals consider fertility preservation before transitioning. Yet only 12% successfully preserve their fertility, due to limited health guidelines^17^. As well, there is almost no collected and reported medical data for monitoring transgender health conditions, with little information on the potential effects of gender affirming hormone therapy on the body^89,90^. Wearables for hormone-based monitoring of gender affirming therapy can allow transgender individuals to feel confident in how their body is transitioning through tangible, accessible data^91^.

For transgender women, undergoing male-to-female transition, estrogen therapy requires the reduction of testosterone from 200 ng/dL (biological male levels) to 75 ng/dL (biological female levels), by intaking estrogen promoting drugs, thereby increasing estrogen and progesterone levels in the body^89,90^. When monitoring male-to-female transition, estrogen hormone therapy is a key factor for wearables design as it can lead to increased risk of disease by which estrogen is an underlying mechanism^92^. Additionally, one of the main side effects of estrogen promoting drugs, such as cyproterone acetate, includes increased depression and hyperprolactinemia^90^.

Outside of hormone therapy, lifestyle factors play a key role in the development of long-term health conditions amongst the transgender population^88,93^. For instance, lifestyle habits such as drinking alcohol, smoking and diet, influences the chances of developing breast cancer for transgender individuals^88,93^. Additionally, gender dysphoria, experienced by transgender women and men, often leads to anxiety, depression and substance abuse, which can in turn affect biometrics like heart rate and sleep patterns^94–97^. As such, a combined biometric and hormonal wearable device that takes gender identity into account would greatly improve experiences of transition^91^. Other challenges to overcome for transgender health technologies are the scarcity of health professionals knowledgeable in transgender health to assist in data interpretation, limited healthcare infrastructure to inform device design and integration into transgender lives, and lack of medical insurance plans supporting gender identity to receive high quality health care^88,91^.

### Wearables tracking abnormal tissues using imaging modalities

For various female-prevalent diseases, the clinical gold standard for diagnostics is imaging abnormal tissue growth or rapid tissue loss^98–100^. For imaging tissue growths amongst women, the breast is an organ of concern as 50% of women develop fibro cysts^98^, and 25% develop benign tumors^100^, referred to as fibroadenomas, over their lifetime. Mammography and ultrasound are the international standard of screening and diagnosing patients^101–103^. Similarly, osteoporosis in women is correlated with low estrogen levels that occur after menopause, resulting in lack of estrogen binding to osteoblasts and increased secretion of cytokines that promote osteoclast differentiation of progenitor cells. Ultimately, this leads to overactivity of bone resorption by high numbers of active osteoclasts (Fig. 1e)^104^. For this objective, women aged over 50 years old, tissue loss particularly in bones, leads to 4$\times$ higher occurrences of osteoporosis compared to men^105^. Regarding bone loss, x-ray-based imaging in hospitals, such as dual-energy x-ray or computed tomography, is the most common diagnostic method^106^.

While effective, these gold standard diagnostic methods are limited in accessibility, relying on highly expensive equipment and healthcare systems^107^. In communities and countries with limited resources and funding for medical equipment, wearable imaging modality technologies, in comparison, provide cost-effective and resource-efficient alternatives^108,109^. Also, early onset diagnostics are limited, but having these conditions determined at early stages can prevent worsening disease symptomology^110^. Additionally, many reports from women indicate feelings of discomfort, fear and anxiety before and after undergoing diagnostic imaging because of prolonged wait times to receive results^107^. To address these concerns, emerging literature points to miniaturizing and integrating of imaging modalities into wearable devices for a faster result^107^.

Sensing antenna development is a promising component of breast imaging wearables^111–113^. Antennas transmit electromagnetic waves (e.g., microwaves, ultrasound, or infrared) into breast tissue, with reflection analyzed to differentiate healthy from malignant tissue based on stiffness and density differences (109–167 kPa for cancerous vs. 10.9–12.4 kPa for normal tissue) (Fig. 2d)^113,114^. While sensing antenna systems can provide full breast imaging, high probe counts (16–512 antennas) complicate user adoption. Recent developments in omnidirectional antennas, which allow simultaneous signal transmission and acquisition, have reduced probe requirements, improving user comfort^115^. Regardless, these systems remain technically complex and require clinical expertise for data interpretation.

Ultrasound breast patches offer better structural flexibility and compatibility with existing ultrasound imaging techniques and have thus been proposed as a preferred alternative for breast diagnostics and screening^115^. Full breast monitoring and deep tissue imaging of cysts as small as 0.3 cm has been reported with ultrasound breast patches composed of crystalline materials, making them a viable option for early-onset detection^115^. An additional advantage of crystal-based patches is their ability to detect electromagnetic waves in the terahertz frequency range, which is safer for patients and more compatible with current clinical workflows than microwave or infrared imaging^116,117^. However, these patches are typically single-use and require clinical evaluation, limiting their application for continuous at-home monitoring^115,116^.

Smart textiles provide a practical solution for everyday breast health monitoring by incorporating wireless sensors into fabrics like cotton, polyester, nylon, and denim (Fig. 2e)^112,118,119^. Textile-based antennas, either stand-alone or embedded into smart bras, can be implemented to measure tumor size and detect tissue abnormalities^120^. These antennas leverage scalable textile manufacturing techniques, such as high-throughput weaving, making them viable for commercial production^121^. Wireless connectivity allows real-time data transmission to smartphone applications, allowing women to monitor their breast health autonomously^119^. Currently, most wearable breast health technologies and smart bras focus exclusively on breast cancer detection. However, conditions such as fibroadenomas and fibrocystic breasts, which involve benign tissue growths, could also benefit from similar wearable monitoring approaches. Future iterations of smart textiles and biosensors should be adapted to differentiate between benign and malignant tissue, expanding their diagnostic and preventative monitoring capabilities^122^.

Imaging and tracking wearables can also help in early management of osteoporosis. Osteoporosis is a musculoskeletal condition characterized by bone mineral density (BMD) loss due to heightened osteoclast activity^123^. The decline in estrogen levels and high potential of developing osteoporosis during menopause accelerates bone resorption, increasing the risk of falls, fracture, sarcopenia and total hip arthroplasty (THA)^124,125^. Gait pattern can be assessed through motion sensors-based mapping and accelerometry, in combination with biometrics such as oximetry and cardiac monitoring, incorporated into wearable devices^126^. Optical polymer fibers in textiles and walking devices track gait through heart rate, respiration, and gait cadence^127^. Meanwhile, in-sole gait monitoring sensors utilizing inertial measurement units (IMUs) offer a discreet, real-time assessment (Fig. 2e)^125^. For postmenopausal women, wearable sensors embedded in textiles or shoes are easily adoptable and can enhance compliance for more accurate monitoring. Regardless, these gait sensors primarily focus on regular walking patterns, with limited data on dynamic movements like exercise or abnormal walking patterns.

Wearables measuring BMD provide a more direct strategy for monitoring osteoporosis compared to gait. For instance, emerging wearable armbands use ultrasonic transduction to measure BMD, detecting femoral ultrasonic velocity (3404.46 m s^−1^) and identifying osteoporosis through T-score testing (T-score ≤ −2.5)^128^. Regardless, BMD abnormalities can also indicate conditions like rheumatoid arthritis, osteoarthritis, and Parkinson’s Disease, requiring BMD monitoring to be paired with electrochemical biosensing to enhance diagnostic precision^129–131^. These wearables, while promising, have not been assessed with large participant populations of elderly, menopausal women, and will require preclinical and clinical trials before being brought to market.

### Wireless wearable technologies for vaginal infection

Vaginitis, or vaginal infection, one of the most common reasons women seek medical attention, impacts sexual and reproductive health^132^. Symptoms include itching, pain, abnormal discharge, and odor, often leading to feelings of embarrassment, anxiety, and reduced quality of life^133,134^. The three most common types of vaginitis are bacterial vaginosis (BV), vulvovaginal candidiasis (VVC), and trichomoniasis^135,136^.

BV, the most frequent vaginal infection among reproductive-age women, is characterized by microbiome imbalances, particularly a loss of *Lactobacilli* and an overgrowth of anaerobic bacteria such as *Gardnerella*, *Prevotella*, and *Mobiluncus*^132,134^. Diagnosis traditionally relies on microscopy-based Nugent scoring and Amsel’s criteria, assessing discharge appearance, odor, and vaginal pH (pH > 4.5). However, these methods suffer from poor sensitivity, inter-observer variability, and reliance on skilled personnel^132^. Similarly, VVC and trichomoniasis are diagnosed through microscopy, and in vitro pH assessment, both of which have limited accuracy^136^. Polymerase Chain Reaction (PCR) diagnostic techniques enhance the specificity and sensitivity of detection by leveraging molecular mechanisms through microorganism DNA detection in biological samples^137^. Regardless, current PCR platforms are only used for clinical and laboratory testing as equipment is costly and requires trained personnel, making it difficult for at-home monitoring^137^.

Research on wearable vaginal infection diagnostics remains limited, underscoring the need for further innovation. Several pH-based biosensors have been developed for BV and VVC detection. For example, intravaginal biosensing rings monitor pH levels continuously using a fluorescent polyelectrolyte-coated porous silica sensor on a PDMS ring^138^. Similarly, a startup company, ALMA, reported the development of biosensing underwear that integrates potentiometric and amperometric sensors into the gusset to track vaginal pH and lactate levels^139^. Additionally, waterproof electronic decals (WPEDs) mounted on tampons measure vaginal fluid pH, transmitting data wirelessly to a smartphone (Fig. 2f)^140^.

These biosensors provide cost-effective, real-time data, but they also face design challenges related to comfort, durability, safety, and hygiene. Issues with adhesion, connectivity, and interference from biological fluids (e.g., menstrual blood) further complicate their adoption. Additionally, pH monitoring alone is insufficient for definitive diagnosis, as abnormal pH is common to both BV and trichomoniasis, necessitating supplementary diagnostic methods^141^.

Beyond pH sensing, innovative biosensors targeting specific vaginal pathogens could enhance diagnostic accuracy. For instance, a thiolated aptamer-based AuNP sensor detects glucans on *Candida albicans*, triggering a colorimetric change from pink to blue upon pathogen detection. Compared to pH-based approaches, this method has increased specificity for VVC^142^. However, its usability may be hindered by both the subjective interpretation of results by non-experts and the complexity of the device’s assembly process, such as calibration and user interface navigation^143^.

Despite advances in BV and VVC detection, wearable diagnostics for trichomoniasis remain unexplored. Expanding biosensor technologies to include pathogen-specific markers and multi-analyte detection could facilitate at-home vaginal health monitoring, offering more accurate, accessible, and user-friendly solutions.

## Portable diagnostics of women-prevalent conditions

Portable diagnostic devices have notable advantages including POC, real-time results, and minimal infrastructure requirements, making them particularly beneficial for women’s health. However, research and development efforts have largely focused on vaginitis and cancer, leaving many other conditions unexplored. Expanding existing POC technology could substantially improve diagnostic accessibility and quality of life. WHO emphasizes that POC devices should align with ASSURED criteria—affordable, sensitive, specific, user-friendly, rapid, equipment-free, and deliverable^144^.

### Point-of-care technologies for vaginal infection detection

Timely and accurate detection of vaginitis is crucial, particularly in pregnancy, to prevent complications^145^. Recent efforts have focused on low-cost microfluidic, paper-based, and electrochemical POC devices. The two most common approaches for vaginal infection detection are enzyme-activity-based and DNA-based methods.

#### Enzyme-activity-based technology for POC devices

**Fig. 3:**
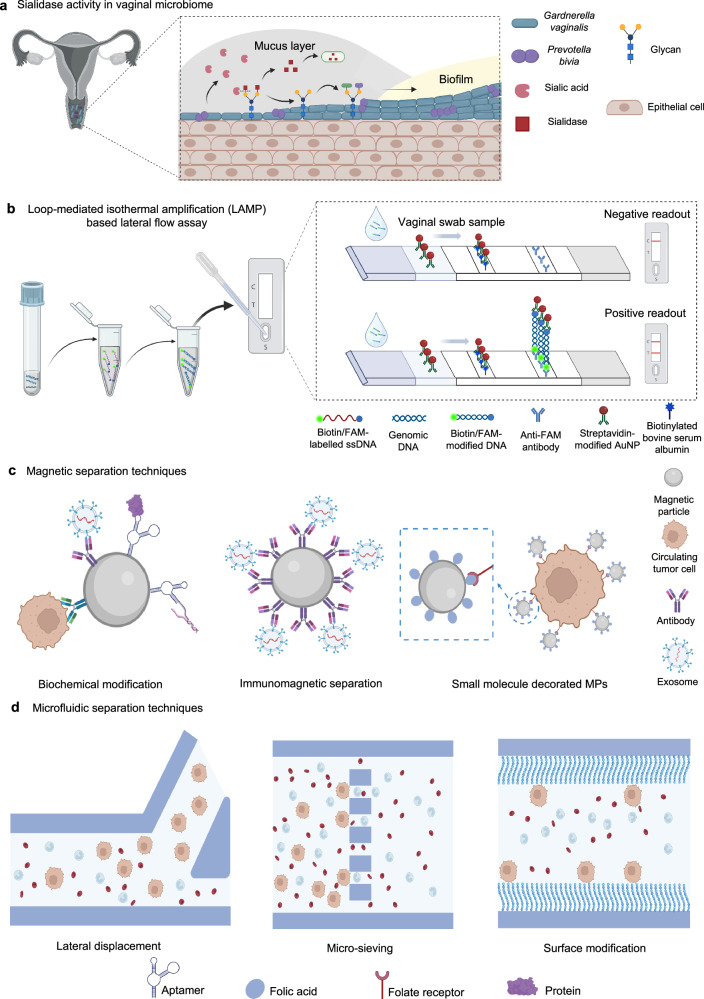
Diagnostic devices for women-prevalent conditions. **a** The mechanism of SLD activity, whereby disialylation of sialoglycon improves bacterial adhesion and biofilm formation. **b** LAMP-based lateral flow strips steps: DNA amplification accrues during the 1 h at isothermal condition; the test line of strip immobilized with anti-FAM antibody; conjugation with biotin/FAM-modified DNA; capturing streptavidin-modified AuNPs; test line signal. Copyright 2022 Frontiers^158^. **c** Biomarker enrichment methods used for breast and gynecological cancer detection. **d** Microfluidic platform technology such as lateral displacement, µ-sieving, and channel surface modifications can be used for enrichment based on physical properties such as size and surface charges. Created in BioRender. Hosseinidoust, Z. (2025) https://BioRender.com/t20f565.

Bacterial enzymes such as vaginolysin (VLY) and sialidase (SLD) are key virulence factors in BV^146^. SLDs, found commonly in vaginal discharge, play a crucial role in cleaving sialic acid from cervicovaginal mucus and epithelial cells, promoting bacterial growth, biofilm formation, and toxin formation (Fig. 3a). Bacterial SLDs also is associated with undesirable pregnancy outcomes and preterm birth^146–149^.

SLD has been the target of numerous colorimetric and fluorescent biosensors. A reported colorimetric biosensor uses 20-(5-bromo-4-chloroindol-3-yl)-a-D-N-acetyl-neuraminic acid (BCIN), which changes color upon contact with vaginal fluid containing SLD^150^. To enhance reaction speed and enzyme absorption, poly(ethyleneimine) is used as a catalyst^150,151^. Although colorimetric biosensors are user-friendly, they often lack quantitative precision. To improve sensitivity, fluorescent SLD detection can be leveraged alongside colorimetric methods. Disposable Y-shaped paper-based biosensors or well-plate sensing platforms incorporate cadmium selenide zinc sulfide (CdSe@ZnS) quantum dots (QDs) or fluorescein isothiocyanate (FITC)-modified antibodies can achieve quantifiable results through fluorescence^148,152^.

#### LAMP-based POC devices

In the diagnosis of VVC, chlamydia trachomatis (CT), and neisseria gonorrhoeae (NG), current gold standard tests include cell culture technology, enzyme-linked immunosorbent assays (ELISAs), nucleic acid amplification tests (NAATs), and PCR-based assays. Of these methods, PCR is the most clinically used as it has >90% sensitivity and specificity^153,154^. These techniques, while effective in clinics, are time consuming, labor some and difficult to incorporate into at-home POC devices.

More accessible POC-compatible alternatives to PCR are loop-mediated isothermal amplification (LAMP) and recombinase polymerase amplification (RPA). LAMP is a rapid, low-cost, and straightforward method for nucleic acid amplification, capable of amplifying the target gene within one hour under an isothermal cycle (50–60 °C) (Fig. 3b)^153^. In comparison, RPA operates at lower temperatures (37–42 °C) and requires fewer primers. Although RPA is more temperature-flexible, LAMP is more widely used due to its resistance to inhibitors, making it ideal for complex clinical samples such as blood and vaginal fluids^153,155,156^.

While lateral flow assays (LFAs) are commonly used in BV POC biosensors, they lack the sensitivity and speed of NAATs^137,157^. To overcome this, LAMP-integrated lateral flow assays (LILFAs) have been developed for multiplex detection of NG and CT. LILFAs utilize biotin- and fluorescein-labeled DNA probes, forming a sandwich-like structure upon target binding, enhancing detection accuracy^158^. However, LILFAs remain qualitative, and further research is needed to incorporate quantitative fluorescence-based detection for bacterial load analysis and infection staging^157,159^.

#### Bacteriophage-based biosensing technologies

Bacteriophage (phage)-based biosensing is an emerging tool for the detection of bacterial pathogens in various settings^160,161^. Phages selectively infect bacteria, injecting genetic material for replication, making them ideal for highly specific bio-probes in electrochemical and optical biosensors^162^. While phage-based diagnostics have been applied in various medical fields, no studies have explored their use in vaginal infection detection. Given their specificity, low-cost, rapid detection, and low detection limits, phage biosensors hold promise for BV diagnostics and multi-pathogen detection in the future^163^.

### Diagnostic technologies for breast and gynecological cancers

Early cancer detection significantly improves survival rates by enabling timely, less invasive treatment^164^. However, 31% of breast cancer and 49% of cervical cancer diagnoses occur at late stages^165^. As a result, five-year survival rates drastically lower, from 93% (stage 1) to 26.9% (stage 3) and 13.4% (stage 4)^166^. Current detection methods—ultrasound, computed tomography, and magnetic resonance imaging (MRI)—lack sensitivity for early-stage diagnosis, are costly, or involve toxic chemicals^164^. POC diagnostics can enhance early biomarker isolation and detection, as summarized in Supplementary Table 2.

#### Enrichment of early-stage women cancer biomarkers

Serological biomarkers are commonly used in immunosensors, ELISAs, and PCR, with key breast and gynecological cancer biomarkers including breast cancer associated gene 1 and 2 (BRCA1 and BRCA2), cancer antigen-125 (CA-125), human epididymis protein 4 (HE-4), mesothelin alpha fetoprotein, osteopontin, human epidermal growth factor receptor 2 (HER2), and human papillomavirus (HPV) virus. However, these markers may overlap with non-cancerous conditions, leading to false negatives^167,168^. For instance, CA-125 is not detectable amongst 50% of women in the first stage of ovarian cancer^169^. Moreover, traditional solid-tissue biopsies are invasive and fail to capture tumor heterogeneity in real time^170^. In comparison, liquid biopsies offer a timely, non-invasive method for cancer detection of a diverse range of biomarkers, found in body fluids such as blood, urine, and saliva^171,172^.

Alternative biomarkers for cancer detection focus on biomolecules secreted by breast and gynecological cancer cells into biological fluids, such as extracellular vehicles (EVs), circulating tumor DNA (ctDNA), microRNA (miRNA), and cancer-derived lipids or proteins. For example, exosomes, ranging from 30 to 200 nm in size, can also be secreted by cancer cells into body fluids at different stages of cancer development depending on the cancer type^141,142^. Additionally, circulating tumor cells (CTCs)—shed from tumors during early metastasis—serve as early-stage cancer biomarkers^173,174^. As all these biomarkers are found in fluid, future diagnostic methods will shift towards liquid biopsies, minimizing the reliance on more invasive solid tumor biopsy, which can severely alter the structure and function of female tissues^175^.

Despite their promise, CTC concentrations in biological fluids are extremely low (1–100 CTCs per million hemocytes), necessitating isolation and enrichment^174^. Conventional methods (flow cytometry, ELISA, ultracentrifugation) are time-consuming, costly, and inefficient for detecting exosomes (<200 nm)^176^. Two key alternative techniques for biomarker isolation with minimal structural disruption and high size resolution are biochemical and biophysical separation^175,177,178^.

Biochemical enrichment relies on biomarker interaction with biorecognition molecules. Magnetic beads (MBs) functionalized with cancer-specific probes enhance biomarker isolation in liquid biopsies. In addition, folate receptors, overexpressed in breast and ovarian cancers, are ideal MB targets for CTC capture (Fig. 3c)^179,180^. However, biochemical methods are limited to singleplex detection, requiring multiple probes for multiplexing^181^. Microfluidic chips, with micrometric channels for single-cell studies, reduce sample volume and costs, making them widely adopted for cancer detection^182,183^.

Biophysical separation, a label-free approach, overcomes biochemical limitations by utilizing filtration, inertial forces, ultrasound, or electrical fields^181^. Recent studies combine size, charge affinity, and surface modifications for enhanced isolation (Fig. 3d)^184,185^. For example, CTCs preferentially adhere to positively charged surfaces, such as lipid or polymer-modified surfaces, leveraging their stronger negative charge compared to normal cells^181,186^. CTCs can also be enriched through filtration or binding to antibody-functionalized polymeric nanoparticles (e.g., polystyrene), then collected downstream in a microfluidic chip^156,157^.

Exosome isolation can also be achieved via surface charge manipulation. Chitosan-coated microfluidic channels, sensitive to pH changes, allow negatively charged exosomes to bind during sample flow. After capture and sample removal, pH modulation releases the exosomes, facilitating collection^186^. This approach can be further enhanced by combining size and charge-based separation^181^.

#### POC technologies for breast and gynecological cancer

Standard cancer diagnostics require trained personnel and hospital-based equipment, limiting accessibility, particularly for women with restricted healthcare access. At-home POC devices, such as LFAs, provide affordable, rapid screening that meets WHO’s ASSURED criteria^141^.

LFAs are paper-based POC devices composed of a sample pad that interacts with the sample fluid and transfers it to the conjugate pad for labeling. The labeled sample moves through the strip by capillary forces, towards the detection pad, modified with aptamers, antibodies and other biomolecules^187^. To increase sample interaction time and increase sensitivity, promising approaches include using magnetic materials, such as probe-modified magnetic particles, which attach to biomarkers on the detection pad and extend interaction time under a magnetic field^188^.

However, conventional LFAs remain primarily qualitative or semi-quantitative, limiting their sensitivity compared to ELISA-based assays. To address sensitivity limitations, luminescent-based dual-mode LFAs incorporate fluorescent microspheres (FMs), QDs, and organic dyes, enabling both visual and quantitative detection^189–191^.

While LFAs effectively detect abundant cancer biomarkers (e.g., CA-125, HER2, HE-4), CTCs and exosomes, due to their low concentrations, require preconcentration before detection. Achieving simultaneous preconcentration, signal amplification, and detection of CTCs and exosomes in LFAs remains a challenge, though emerging strategies have been reported. Pen-based LFAs, using a catalyzed hairpin assembly (CHA) technique, are especially promising in their ease of use and all-in-one design^192^.

#### Multiplex detection for women-prevalent cancers

Relying on a single biomarker for cancer detection can lead to high false-positive rates, as some biomarkers for women’s cancers are associated with other women’s conditions. For example, CA-125, a key ovarian cancer biomarker, is also elevated during endometriosis^193–195^. Similarly, breast cancer subtypes—HER2-positive, Luminal A/B, and Basal-like—require distinct biomarker panels (HER2, estrogen receptor (ER), and progesterone receptor (PR)) to guide treatment selection^196,197^.

Multiplex biosensors enhance diagnostic specificity by detecting multiple cancer biomarkers simultaneously. For instance, miRNA profiles (miR-21, miR-106a, miR-155) vary across breast cancer subtypes, necessitating multiplex detection strategies^198^. Electrochemical biosensors are well-suited for the development of flexible sensors due to fast response, inherent miniaturization, convenient operation, portability, and integration of cost-effective electrodes. Unlike optical methods, electrochemical biosensors provide direct electrical readouts, enabling real-time biomarker analysis in complex biological samples^199^. Enzyme-based electrochemical (EBEC) and electrochemiluminescence (ECL) biosensors enhance signal amplification and sensitivity, which can be achieved by labeling the probes with redox species such as methylene blue, horseradish peroxidase (HRP), and metal ions (e.g., gold, silver) for EBEC, or with fluorescent labels for ECL^200,201^.

Multiplex LFA-based POC devices are particularly relevant for ovarian cancer, one of the most difficult cancers to detect in its early stages. Since HE-4 levels in urine vary depending on hydration status and urine concentration, creatinine levels are used as a normalizer for urinary biomarkers^193,202^. It is also essential to measure the HE-4/creatinine ratio for detecting ovarian cancer in urine. The cut-off value for HE-4 is 3.5, with the ratio range being around 2 for healthy individuals and up to 45 for late-stage cancer patients. For this purpose, a dual LFA system has been reported with signals quantified using a smartphone and scanner^195^. One strip was designed to detect HE-4 using a sandwich-like system with antibodies and antibody-modified gold shells as reporters, while the other strip was designed for creatinine detection through a series of chemical reactions.

Despite advancements, few LFAs are available for detecting multiple protein biomarkers. LFAs sensitivity, in both singleplex and multiplex detection, is often limited by the hook effect that prevents the formation of the antibody-antigen complex^203^. This leads to false-negative results when targeting more than two protein biomarkers at high concentrations. Vertical flow assays overcome this limitation. Surface-enhanced Raman spectroscopy (SERS)-vertical flow assays have shown promise for the multiplex detection of exosome proteins (Mucin short variant S1 (MUC1), HER2, and CEA) which are useful for identifying breast cancer subtypes. This vertical flow assay contains a single aptamer-modified test pad that captures each biomarker on a specific spot, followed by detection using SERS probes that offer ultrasensitive, non-destructive analysis capabilities and minimal photobleaching^204^.

## Commercialization, barriers to entry and future scopes

Recent advancements in women’s health technologies–particularly in wearable biosensors and POC diagnostic devices–have accelerated the transition from conceptualization to clinical trials, driving increased stakeholder investment and introduction of these technologies into international markets (Supplementary Table 3)^205^.

### Barriers to entry of women’s wearable technologies

A recent surge in technologies in the commercial space of women’s health have advanced significantly in the product pipeline for new product development (NPD)^205^. For instance, a majority of fertility wearable tracking devices are readily available for clinicians, researchers and consumers to purchase and use^206–210^. Additionally, companies such as Dexcom and Freestyle Libre have led the development of continuous glucose monitoring wearables in the commercial space^211,212^. For highly specialized devices, such as those targeting women-prevalent cancer screening and osteoporosis monitoring, these technologies are not as advanced in the product development pipeline, with many going through clinical trials or the early stages of product scale up^213–220^. The success of these commercial devices relies on ease of integration into the currently existing infrastructure, where devices designed to be compatible with current medical best practices and mHealth applications have a higher chance of sucess^221,222^. On the other hand, devices that are costly in terms of manufacturing and materials are more likely to fail^221,222^. Women’s health is a rapidly growing commercial space, therefore intellectual property in the field is expected to grow and thus patenting new technologies becomes even more important for technologies geared towards commercialization^223^. Bringing women’s health technologies from prototype to commercialization involves multiple challenges. This section highlights key barriers in usability, adoption, and policy-making that impact market success.

#### Usability, adoption and compliance for diverse women

Women’s health technologies in commercialized spaces are most successful when they are designed for diverse populations of cisgender women and transgender people from different socioeconomic and cultural communities^224,225^. Age is also essential to consider when designing user interfaces for monitoring and diagnosing disease associated with menopause. Digital knowledge gaps may restrict usability for older generations of women^226,227^. Meeting these considerations can ensure compliance and autonomy when using digital devices^228^.

Furthermore, women from low socioeconomic status or enfranchised communities adopt biomonitoring technologies less than women of high socioeconomic status^229^. The reason for this is the lack of access to digital health platforms and internet needed for device function, as well as limited opportunities to partake in educational or policy decision making initiatives^62^. Often, the voices of these women are not heard on the world political stage, and these women do not have expectations that wearable devices can be designed for them, creating a market barrier in a similar way as differences in age. This is because of poverty, cultural disparities and income inequality, as well as home or job insecurity^62,230,231^. These experiences are exacerbated amongst transgender individuals, who are over twice as likely to experience low socioeconomic conditions compared to the cisgender population in the US^232–234^.

#### Global policy making for women’s health technologies

One challenge in the development of technologies is the lack of global policy making practices for women’s health. Within the past decade, a large positive shift towards the globalization of women’s health and sexual rights has taken place. On an international front, the United Nations (UN) and WHO acknowledged the lack of policy making and women’s sexual rights laws which has limited women’s access to healthcare and medical devices^235,236^. Many international governments have taken initiatives and measures to remove gender and sex discrimination, and promote women’s self-autonomy, medical education and access to healthcare resources. This includes the United States (e.g., National Institutes of Health’s (NIH’s) Women Health Initiative), Canada (e.g., Health Canada’s National Women’s Health Research Initiative), Europe (e.g., European Union (EU) Manifesto for Women’s Health), and Australia (e.g., Australia’s National Women’s Health Strategy 2020–2030)^237–241^. Additionally, international organizations, such as United Nations (e.g., United Nations Population Fund) and WHO (e.g., Global Strategy for Women’s, Children’s and Adolescents’ Health), are collaborating with countries in Africa, the Middle East, and Asia to initiate women’s health and equality policies by 2030^242,243^.

### Artificial intelligence in women’s health technologies

From a clinical perspective, AI and ML can be leveraged to significantly improve predictive capabilities and offer dynamic pattern recognition to enhance early-onset diagnostics, automate data collection and streamline care^244^. Examples of AI and ML algorithms of interest include convolutional neural networks (CNNs)^245^, artificial neural networks (ANNs)^18^, support vector machine (SVM)^246^, multivariate logistic regression (LR), random forest (RF), extreme gradient boosting (XGBoost), and light gradient boosting machine (LightGBM)^247^. In women’s health, AI and ML algorithms can enhance health outcomes for women facing pregnancy complications (e.g., preterm births, spontaneous abortion, GDM, preeclampsia, neonatal deaths, congenital anomalies)^18,246^, vaginal infections^245,248^, and cancers^249–256^ (Supplementary Table 4).

In diagnostics, new biomarker discovery using AI can introduce unique or more all-encompassing diagnostic strategies by providing more holistic understanding of disease mechanisms for conditions affecting women, thus bridging the knowledge gap^246^. An example can be identifying new set of biomarkers in urine for determining GDM that affects many organ systems of pregnant women and has multiple disease mechanisms for different symptomologies^246,257^. AI and ML algorithms can also collect and compile a wide range of predictive values simultaneously, including EHG, metabolic biomarker levels, ultrasound images of abdomen and cervix, and demographic profiles, to predict complication occurrences in the first trimester^246^. Additionally, AI-powered mHealth applications offer a valuable tool for assessing early symptoms and recommending timely interventions, an example includes AI-powered smart phone tools using MGH Perinatal Depression Scale (MGHPDS) for postpartum depression, which affects approximately 15% of mothers^258,259^. Identifying diseases by comparing hundreds of sample images is another capability, as AI and ML algorithms can quickly pick up discrepancies that can be missed by the human eye. For instance, CNNs can detect the presence of yeast hyphae from microscopy images for detecting VVC, exceeding expert diagnostics by 4.58%^245^.

Some mHealth applications and wearable technologies that leverage AI and ML have already been commercialized for women’s health applications or are close to commercialization^260^. For example, the OvuSense platform, which has been incorporated into viO’s OvuFirst device, is a commercial device that obtains temperature data for fertility monitoring and utilizes algorithms for improved fertility predictions^261^. Copan Diagnostics has also developed and released PhenoMatrix to detect multiple vaginal infections by comparing images of vaginal swab plate cultures^248^. Select studies have shown how AI has been implemented for improving accuracy of cervical and breast cancer diagnosis; these technologies have received Food and Drug Administration (FDA) regulatory approval for commercial use^249–256^. One example is General Electric’s FDA approved AI-powered breast cancer prediction tool based on ultrasound data^262^.

In applying AI and ML for clinical practice, there is a dire need to understand the effects of generalized data and how we can shift away from it for reliable outputs^263^. An untapped use of AI is in assessing the possibility of undetermined or undetected biological sex differences amongst diseases that affect both males and females^264^. One example is the use of AI-powered statistical analyses comparing women with and without menstrual cycles, as well as males, to determine the variance of body temperature to overcome sex biases by including equal data on females and males^265^. If trained on unbiased data sets, AI and ML have the power to distinguish factors that lead to predominance of diseases in women compared to men, such as CVD and osteoporosis^264^. These algorithms also have the potential to identify other unknown conditions that predominantly affect women, providing insight and knowledge into unidentified health gaps^264^. However, the main challenge in algorithm development is that they typically leverage historically reported biased medical data and biomedical research as part of their training processes^264,266^. As mentioned, a large biological sex bias and gender identity disparity exists in literature and medical records, which could translate to the model after training^264,267^. This barrier to entry for emerging AI and ML models necessitates the need for further biomedical research addressing this biological sex bias. It also highlights the importance of reporting unbiased data that includes equal representation of all sexes, genders and backgrounds^268^.

## Outlook

This review highlights key bioengineering advancements in women’s health diagnostics and biomonitoring. As more prototypes and in vivo studies emerge, the field is progressing toward commercialization, transforming economic, healthcare, and societal perceptions of women’s health monitoring and diagnostics. One outstanding challenge in these emerging technologies is the limited data from underrepresented populations to inform device design and user interface accessibility. To ensure equitable healthcare solutions, future biomedical devices must be inclusive, addressing the needs of cisgender women and transgender individuals across diverse backgrounds.

As more wearable technologies and detection platforms for women’s health are created, integrating wearable technologies with ML and AI will not only enhance detection accuracy but also reduce user error in data interpretation. AI-driven systems can personalize diagnostics, ensuring precise monitoring of cancers, infections, and reproductive health conditions. AI algorithms can predict bacterial imbalances before a full-blown infection develops, allowing for earlier intervention^269^. Similarly, in cancer detection, AI can analyze biomarker fluctuations, predict cancer progression, metastasis risk, and immune responses (e.g., NK cell activation), leading to earlier and more targeted treatments.

The future of women’s health technologies is shifting toward wireless, AI-integrated wearables that provide real-time predictive feedback, enhanced user interfaces, and seamless remote monitoring. These innovations will assist women by enabling personalized, proactive healthcare management. By bridging bioengineering, AI, and digital health, the next generation of wearable diagnostics will transform women’s health monitoring into a more accessible, data-driven, and patient-centered paradigm.

## Supplementary information

Supplementary Information

## References

[1] Shaw, L. J. et al. Quality and equitable health care gaps for women: attributions to sex differences in cardiovascular medicine. *J. Am. College Cardiol.* **70**, 373–388 (2017). 10.1016/j.jacc.2017.05.051 28705320

[2] Benyamini, Y. & Todorova, I. Women’s Reproductive Health in Sociocultural Context. *Int. J. Behav. Med.* **24**, 799–802 (2017). 10.1007/s12529-017-9695-7 29150752

[3] Sutton, M. Y., Anachebe, N. F., Lee, R. & Skanes, H. Racial and ethnic disparities in reproductive health services and outcomes, 2020. *Obstet. Gynecol.* **137**, 225–233 (2021). 10.1097/AOG.0000000000004224 PMC7813444 33416284

[4] Women’s health got worse in 2021, global survey finds. https://www.cnn.com/2022/09/21/health/global-womens-health-index-2021/index.html (2022).

[5] Samson Enitan, S. Improving women’s health in the 21st century: current challenges, medical advancements and future prospects. *Women Health Care Issues* **6**, 01–07 (2023).

[6] Magnus, M. et al. Development of a telehealth intervention to promote care-seeking among transgender women of color in Washington, DC. *Public Health Nurs.* **37**, 262–271 (2020). 32017202 10.1111/phn.12709

[7] White, J. & Clayton, J. The gender health innovation gap: a perspective from the NIH Office of Research on Women’s Health. *Med.* **3**, 298–301 (2022). 10.1016/j.medj.2022.04.010 35584651

[8] Merone, L., Tsey, K., Russell, D. & Nagle, C. Sex inequalities in medical research: a systematic scoping review of the literature. *Women’s Health Rep.* **3**, 49–59 (2022). 10.1089/whr.2021.0083 PMC8812498 35136877

[9] Lyzwinski, L., Elgendi, M. & Menon, C. Innovative approaches to menstruation and fertility tracking using wearable reproductive health technology: systematic review. *J. Med. Internet Res.* **26**, e45139 (2024). 10.2196/45139 PMC10905339 38358798

[10] Zuo, X., Yang, X., Dou, Z. & Wen, J. R. RUCIR at TREC 2019: conversational assistance track. In *28th Text REtrieval Conference, TREC 2019 - Proceedings* (National Institute of Standards and Technology (NIST), 10.1145/1122445.1122456 2019).

[11] Common chronic diseases in women compared to men. https://www.canada.ca/en/public-health/services/publications/diseases-conditions/common-chronic-diseases-women-compared-men-aged-65-years-older.html (2021).

[12] WHO. Ten top issues for women’s health. https://www.who.int/news-room/commentaries/detail/ten-top-issues-for-women’s-health (2015).

[13] Singh, V. AI, women’s health care, and trust: problems and prospects. *Artif. Intell. Mach. Learn. Women’s Health Issues* 235–254 10.1016/B978-0-443-21889-7.00002-6 (2024).

[14] Pan, M. et al. Effects of wearable physical activity tracking for breast cancer survivors: a systematic review and meta-analysis. *Int J. Nurs. Knowl.* **35**, 117–129 (2024). 36843066 10.1111/2047-3095.12418

[15] Dhingra, L. S. et al. Use of wearable devices in individuals with or at risk for cardiovascular disease in the US, 2019 to 2020. *JAMA Netw. Open* **6**, e2316634 (2023). 37285157 10.1001/jamanetworkopen.2023.16634 PMC10248745

[16] Mattison, G. et al. The influence of wearables on health care outcomes in chronic disease: systematic review. *J. Med Internet Res.* **24**, e36690 (2022). 35776492 10.2196/36690 PMC9288104

[17] Leung, A., Sakkas, D., Pang, S., Thornton, K. & Resetkova, N. Assisted reproductive technology outcomes in female-to-male transgender patients compared with cisgender patients: a new frontier in reproductive medicine. *Fertil. Steril.* **112**, 858–865 (2019). 31594633 10.1016/j.fertnstert.2019.07.014

[18] Togunwa, T. O., Babatunde, A. O. & Abdullah, K.-R. Deep hybrid model for maternal health risk classification in pregnancy: synergy of ANN and random forest. *Front. Artif. Intell.* **6**, 1213436 (2023). 10.3389/frai.2023.1213436 PMC10354509 37476504

[19] World Health Organization. More than a third of women experience lasting health problems after childbirth, new research shows. *World Health Organization* https://www.who.int/news/item/07-12-2023-more-than-a-third-of-women-experience-lasting-health-problems-after-childbirth (2023).

[20] World Health Organization. Maternal mortality. World Health Organization https://www.who.int/news-room/fact-sheets/detail/maternal-mortality (2025).

[21] Goodale, B. M. et al. Wearable sensors reveal menses-driven changes in physiology and enable prediction of the fertile window: observational study. *J. Med Internet Res.* **21**, e13404 (2019). 30998226 10.2196/13404 PMC6495289

[22] Shilaih, M. et al. Modern fertility awareness methods: wrist wearables capture the changes in temperature associated with the menstrual cycle. *Biosci. Rep.* **38**, BSR20171279 (2018). 10.1042/BSR20171279 PMC6265623 29175999

[23] Vanmarkenlichtenbelt, W. et al. Evaluation of wireless determination of skin temperature using iButtons. *Physiol. Behav.* **88**, 489–497 (2006). 16797616 10.1016/j.physbeh.2006.04.026

[24] Garcia, A. M. C. et al. Luteal phase of the menstrual cycle increases sweating rate during exercise. *Braz. J. Med. Biol. Res.* **39**, 1255–1261 (2006). 16981051 10.1590/s0100-879x2006005000007

[25] Upton, T. J. et al. High-resolution daily profiles of tissue adrenal steroids by portable automated collection. *Sci. Transl. Med.* **15**, eadg8464 (2023). 37343084 10.1126/scitranslmed.adg8464

[26] myHealth Alberta. Basal Body Temperature (BBT) Tracking. *Government of Alberta* https://myhealth.alberta.ca/Health/pages/conditions.aspx?hwid=hw202058 (2024).

[27] Steward, K. & Raja, A. *Physiology, Ovulation and Basal Body Temperature*. https://www.ncbi.nlm.nih.gov/books/NBK546686/ (2023). 31536292

[28] Williams, A. M. FAM basics: basal body temperature (BBT). *Nat. Womanhood* https://naturalwomanhood.org/fertility-awareness-method-basics-basal-body-temperature-bbt/?utm_source=chatgpt.com (2021).

[29] Wei, L. et al. Wearable sweat management technologies. *Adv. Mater Technol.* **9**, 2470031 (2024).

[30] Charkoudian, N. & Stachenfeld, N. Sex hormone effects on autonomic mechanisms of thermoregulation in humans. *Auton. Neurosci.* **196**, 75–80 (2016). 26674572 10.1016/j.autneu.2015.11.004

[31] Baker, F. C., Siboza, F. & Fuller, A. Temperature regulation in women: effects of the menstrual cycle. *Temperature* **7**, 226–262 (2020). 10.1080/23328940.2020.1735927 PMC7575238 33123618

[32] Zhang, Z., DiVittorio, J. R., Joseph, A. M. & Correa, S. M. The effects of estrogens on neural circuits that control temperature. *Endocrinology* **162**, bqab087 (2021). 33939822 10.1210/endocr/bqab087 PMC8237993

[33] Forman, R. G., Chapman, M. C. & Steptoe, P. C. The effect of endogenous progesterone on basal body temperature in stimulated ovarian cycles. *Hum. Reprod.* **2**, 631–634 (1987). 3125209 10.1093/oxfordjournals.humrep.a136605

[34] Evans-Hoeker, E. et al. Cervical mucus monitoring prevalence and associated fecundability in women trying to conceive. *Fertil. Steril.* **100**, 1033–1038.e1 (2013). 23850303 10.1016/j.fertnstert.2013.06.002 PMC3787999

[35] Boyd, P. et al. A temperature-monitoring vaginal ring for measuring adherence. *PLoS One* **10**, e0125682 (2015). 25965956 10.1371/journal.pone.0125682 PMC4429109

[36] Papaioannou, S., Aslam, M., Al Wattar, B. H., Milnes, R. C. & Knowles, T. G. User’s acceptability of OvuSense: a novel vaginal temperature sensor for prediction of the fertile period. *J. Obstet. Gynaecol. (Lahore)* **33**, 705–709 (2013). 10.3109/01443615.2013.817984 24127960

[37] Keeler Bruce, L., González, D., Dasgupta, S. & Smarr, B. L. Biometrics of complete human pregnancy recorded by wearable devices. *NPJ Digit Med.* **7**, 207 (2024). 39134787 10.1038/s41746-024-01183-9 PMC11319646

[38] Tarvonen, M. et al. Intrapartum cardiotocography with simultaneous maternal heart rate registration improves neonatal outcome. *Am. J. Obstet. Gynecol.* **230**, 379.e1–379.e12 (2024). 38272284 10.1016/j.ajog.2024.01.011

[39] Afari, H. A., Davis, E. F. & Sarma, A. A. Echocardiography for the pregnant heart. *Curr. Treat. Options Cardiovasc. Med.* **23**, 55 (2021). 34075291 10.1007/s11936-021-00930-5 PMC8160078

[40] Rosen, H. & Yogev, Y. Assessment of uterine contractions in labor and delivery. *Am. J. Obstet. Gynecol.* **228**, S1209–S1221 (2023). 37164494 10.1016/j.ajog.2022.09.003

[41] Leeners, B. Utilizing wearable biosensor technology for monitoring sleep duration patterns in pregnancy – a pilot study. *Endocrinol. Disord.* **8**, 01–10 (2024).

[42] Ryu, D. et al. Comprehensive pregnancy monitoring with a network of wireless, soft, and flexible sensors in high-and low-resource health settings. *Proc. Natl. Acad. Sci. USA* **118**, e2100466118 (2021). 33972445 10.1073/pnas.2100466118 PMC8157941

[43] Aggarwal, G. & Wei, Y. Non-invasive fetal electrocardiogram monitoring techniques: potential and future research opportunities in smart textiles. *Signals* **2**, 392–412 (2021).

[44] Mongan, W. et al. A multi-disciplinary framework for continuous biomedical monitoring using low-power passive RFID-based wireless wearable sensors. *2016 IEEE International Conference on Smart Computing, SMARTCOMP 2016*10.1109/SMARTCOMP.2016.7501674 (2016).

[45] Phillips, N. E. et al. The metabolic and circadian signatures of gestational diabetes in the postpartum period characterised using multiple wearable devices. *Diabetologia* **68**, 419–432 (2025). 39531039 10.1007/s00125-024-06318-x PMC11732869

[46] Wei, H. X., Yang, Y. L., Luo, T. Y. & Chen, W. Q. Effectiveness of mobile health interventions for pregnant women with gestational diabetes mellitus: a systematic review and meta-analysis. *J. Obstet. Gynaecol. (Lahore)* **43**, 2245906 (2023). 10.1080/01443615.2023.2245906 37605977

[47] Kytö, M. et al. Behavior change app for self-management of gestational diabetes: design and evaluation of desirable features. *JMIR Hum. Factors* **9**, e36987 (2022). 36222806 10.2196/36987 PMC9607927

[48] Cheung, N. W. et al. A pilot randomised controlled trial of a text messaging intervention with customisation using linked data from wireless wearable activity monitors to improve risk factors following gestational diabetes. *Nutrients* **11**, 590 (2019). 30862052 10.3390/nu11030590 PMC6470941

[49] Kytö, M. et al. Comprehensive self-tracking of blood glucose and lifestyle with a mobile application in the management of gestational diabetes: a study protocol for a randomised controlled trial (eMOM GDM study). *BMJ Open* **12**, e066292 (2022). 36344008 10.1136/bmjopen-2022-066292 PMC9644362

[50] Kytö, M. et al. Supporting the management of gestational diabetes mellitus with comprehensive self-tracking: mixed methods study of wearable sensors. *JMIR Diabetes* **8**, e43979 (2023). 37906216 10.2196/43979 PMC10646680

[51] Penders, J., Altini, M., Van Hoof, C. & Dy, E. Wearable sensors for healthier pregnancies. *Proc. IEEE* **103**, 179–191 (2015).

[52] Jin, Y. et al. Enhanced detection of Cystatin C for predicting adverse outcomes in gestational diabetes mellitus using a point-of-care immunosensor. *Bioelectrochemistry* **163**, 108907 (2025). 39823996 10.1016/j.bioelechem.2025.108907

[53] Ehrlich, S. F. et al. Using a consumer-based wearable activity tracker for physical activity goal setting and measuring steps in pregnant women with gestational diabetes mellitus: exploring acceptance and validity. *BMC Pregnancy Childbirth* **21**, 420 (2021). 34103002 10.1186/s12884-021-03900-8 PMC8188700

[54] Karnain Wadoo, O., Ahmad, I. & Sayeed, S. I. Reduced lung function and progression to prediabetes: a prospective study. *Iran. J. Diabetes Obes.*10.18502/ijdo.v13i4.7994 (2021).

[55] Tehrani, F. et al. An integrated wearable microneedle array for the continuous monitoring of multiple biomarkers in interstitial fluid. *Nat. Biomed. Eng.* **6**, 1214–1224 (2022). 35534575 10.1038/s41551-022-00887-1

[56] Teymourian, H. et al. Microneedle-based detection of ketone bodies along with glucose and lactate: toward real-time continuous interstitial fluid monitoring of diabetic ketosis and ketoacidosis. *Anal. Chem.* **92**, 2291–2300 (2020). 31874029 10.1021/acs.analchem.9b05109

[57] Chen, Y. et al. A gold nanoparticles and mxene nanocomposite based electrochemical sensor for point-of-care monitoring of serum biomarkers. *ACS Nano* **19**, 16980–16994 (2025). 40262058 10.1021/acsnano.5c03194

[58] Smyth, S., Curtin, E., Tully, E., Molphy, Z. & Breathnach, F. Smartphone apps for surveillance of gestational diabetes: scoping review. *JMIR Diabetes* **7**, e38910 (2022). 36409549 10.2196/38910 PMC9723973

[59] Asadollahi, F., Zagami, S. E., Eslami, S. & Roudsari, R. L. Barriers and facilitators for mHealth utilization in pregnancy care: a qualitative analysis of pregnant women and stakeholder’s perspectives. *BMC Pregnancy Childbirth* **25**, 141 (2025). 39934681 10.1186/s12884-025-07244-5 PMC11817079

[60] Walter, J. R. et al. The future of remote monitoring for pregnancy. *Bridge (Wash. D. C.)* **52**, 16 (2022). 38111590 PMC10727511

[61] Veinot, T. C., Mitchell, H. & Ancker, J. S. Good intentions are not enough: how informatics interventions can worsen inequality. *J. Am. Med. Inform. Assoc.* **25**, 1080–1088 (2018). 29788380 10.1093/jamia/ocy052 PMC7646885

[62] Girmay, M. Digital health divide: opportunities for reducing health disparities and promoting equitable care for maternal and child health populations. *Int. J. Matern. Child Health AIDS* **13**, e026 (2024). 10.25259/IJMA_41_2024 PMC11705165 39776789

[63] Avila-Varela, D. S. et al. Whole-brain dynamics across the menstrual cycle: the role of hormonal fluctuations and age in healthy women. *npj Women’s Health* **2**, 8 (2024).

[64] Kumar, P. & Sait, S. Luteinizing hormone and its dilemma in ovulation induction. *J. Hum. Reprod. Sci.* **4**, 2–7 (2011). 10.4103/0974-1208.82351 PMC3136063 21772731

[65] Lee, Y. & Gao, W. Non-invasive hormone monitoring with a wearable sweat biosensor. *Nat. Rev. Bioeng.*10.1038/s44222-025-00276-8 (2025).

[66] Ye, C. et al. A wearable aptamer nanobiosensor for non-invasive female hormone monitoring. *Nat. Nanotechnol.* **19**, 330–337 (2023). 37770648 10.1038/s41565-023-01513-0 PMC10954395

[67] Altindag, O. et al. Relation of cortisol levels and bone mineral density among premenopausal women with major depression. *Int. J. Clin. Pract.* **61**, 416–420 (2007). 17313608 10.1111/j.1742-1241.2006.01276.x

[68] Qiao, L., Benzigar, M. R., Subramony, J. A., Lovell, N. H. & Liu, G. Advances in sweat wearables: sample extraction, real-time biosensing, and flexible platforms. *ACS Appl Mater. Interfaces* **12**, 34337–34361 (2020). 32579332 10.1021/acsami.0c07614

[69] Lee, H. B., Meeseepong, M., Trung, T. Q., Kim, B. Y. & Lee, N. E. A wearable lab-on-a-patch platform with stretchable nanostructured biosensor for non-invasive immunodetection of biomarker in sweat. *Biosens. Bioelectron.* **156**, 112133 (2020). 32174559 10.1016/j.bios.2020.112133

[70] Li, Z., Chen, F., Zhu, N., Zhang, L. & Xie, Z. Tip-enhanced sub-femtomolar steroid immunosensing via micropyramidal flexible conducting polymer electrodes for at-home monitoring of salivary sex hormones. *ACS Nano* **17**, 21935–21946 (2023). 37922489 10.1021/acsnano.3c08315

[71] Sekar, M., Pandiaraj, M., Bhansali, S., Ponpandian, N. & Viswanathan, C. Carbon fiber based electrochemical sensor for sweat cortisol measurement. *Sci. Rep.* **9**, 1–14 (2019). 30674991 10.1038/s41598-018-37243-w PMC6344552

[72] Zheng, J., Feng, Q., Zheng, S. & Xiao, X. Maternal nutrition and the developmental origins of osteoporosis in offspring: Potential mechanisms and clinical implications. *Exp. Biol. Med.* **243**, 836–842 (2018). 10.1177/1535370218779024 PMC6022911 29792069

[73] Madigan, J. A. et al. Perinatal hair cortisol concentrations linked to psychological distress and unpredicted birth complications. *Psychoneuroendocrinology* **161**, 106921 (2024). 38141367 10.1016/j.psyneuen.2023.106921

[74] Stirrat, L. I. et al. Pulsatility of glucocorticoid hormones in pregnancy: Changes with gestation and obesity. *Clin. Endocrinol. (Oxf.)* **88**, 592–600 (2018). 29314170 10.1111/cen.13548 PMC5887976

[75] Hassan, S., Muere, A. & Einstein, G. Ovarian hormones and chronic pain: a comprehensive review. *Pain* **155**, 2448–2460 (2014). 25172822 10.1016/j.pain.2014.08.027

[76] Chantalat, E. et al. Estrogen receptors and endometriosis. *Int. J. Mol. Sci.* **21**, 2815 (2020). 32316608 10.3390/ijms21082815 PMC7215544

[77] Patel, S. Polycystic ovary syndrome (PCOS), an inflammatory, systemic, lifestyle endocrinopathy. *J. Steroid Biochem Mol. Biol.* **182**, 27–36 (2018). 29678491 10.1016/j.jsbmb.2018.04.008

[78] Hussain, S. M., Cicuttini, F. M., Alyousef, B. & Wang, Y. Female hormonal factors and osteoarthritis of the knee, hip and hand: a narrative review. *Climacteric* **21**, 132–139 (2018). 29378442 10.1080/13697137.2017.1421926

[79] Pérez-López, F. R., Larrad-Mur, L., Kallen, A., Chedraui, P. & Taylor, H. S. Gender differences in cardiovascular disease: hormonal and biochemical influences. *Reprod. Sci.* **17**, 511–531 (2010). 20460551 10.1177/1933719110367829 PMC3107852

[80] Chakraborty, S., Ganti, A. K., Marr, A. & Batra, S. K. Lung cancer in women: role of estrogens. *Expert Rev. Respir. Med.* **4**, 509–518 (2010). 20658912 10.1586/ers.10.50 PMC2928145

[81] Meng, X., Li, Z., Yue, W., Zhang, L. & Xie, Z. Toward at-home and wearable monitoring of female hormones: emerging nanotechnologies and clinical prospects. *ACS Sens.*10.1021/acssensors.4c02877 (2025). 10.1021/acssensors.4c02877 39761986

[82] DiVasta, A. D. et al. Hormonal add-back therapy for females treated with gonadotropin-releasing hormone agonist for endometriosis. *Obstet. Gynecol.* **126**, 617–627 (2015). 26181088 10.1097/AOG.0000000000000964 PMC4545413

[83] Karakas, S. E. New biomarkers for diagnosis and management of polycystic ovary syndrome. *Clin. Chim. Acta* **471**, 248–253 (2017). 28624501 10.1016/j.cca.2017.06.009

[84] Constantin, A. Estradiol in systemic lupus erythematosus. *Acta Endocrinol. (Buchar.)* **19**, 274–276 (2023). 37908893 10.4183/aeb.2023.274 PMC10614577

[85] Xiang, D., Liu, Y., Zhou, S., Zhou, E. & Wang, Y. Protective effects of estrogen on cardiovascular disease mediated by oxidative stress. *Oxid. Med. Cell Longev.* **2021**, 5523516 (2021). 10.1155/2021/5523516 PMC8260319 34257804

[86] Javed, A. et al. The relationship between myocardial infarction and estrogen use: a literature review. *Cureus*10.7759/cureus.46134 (2023). 10.7759/cureus.46134 PMC10612533 37900417

[87] Reisner, S. L. et al. Global health burden and needs of transgender populations: a review. * Lancet* **388**, 412–436 (2016). 27323919 10.1016/S0140-6736(16)00684-X PMC7035595

[88] Safer, J. D. et al. Barriers to healthcare for transgender individuals. *Curr. Opin. Endocrinol. Diabetes Obes.* **23**, 168–171 (2016). 26910276 10.1097/MED.0000000000000227 PMC4802845

[89] Tangpricha, V. & den Heijer, M. Oestrogen and anti-androgen therapy for transgender women. *Lancet Diabetes Endocrinol.* **5**, 291 (2016). 27916515 10.1016/S2213-8587(16)30319-9 PMC5366074

[90] Sudhakar, D., Huang, Z., Zietkowski, M., Powell, N. & Fisher, A. R. Feminizing gender-affirming hormone therapy for the transgender and gender diverse population: an overview of treatment modality, monitoring, and risks. *Neurourol. Urodyn.* **42**, 903–920 (2023). 36403287 10.1002/nau.25097

[91] Haimson, O. L., Gorrell, D., Starks, D. L. & Weinger, Z. Designing trans technology. In *Proceedings of the 2020 CHI Conference on Human Factors in Computing Systems* 1–13 (ACM, New York, NY, USA, 2020). 10.1145/3313831.3376669.

[92] Johansson, T. et al. Contemporary menopausal hormone therapy and risk of cardiovascular disease: Swedish nationwide register based emulated target trial. *BMJ* e078784. 10.1136/bmj-2023-078784 (2024). 10.1136/bmj-2023-078784 PMC11600536 39603704

[93] Kidd, J. D. et al. Prevalence of substance use and mental health problems among transgender and cisgender U.S. adults: Results from a national probability sample. *Psychiatry Res.* **326**, 115339 (2023). 37429172 10.1016/j.psychres.2023.115339 PMC10528335

[94] Berliere, M. et al. Effects of hormones on breast development and breast cancer risk in transgender women. *Cancers* **15**, 245 (2023). 10.3390/cancers15010245 PMC9818520 36612241

[95] Heng, Y. J. et al. Effect of testosterone therapy on breast tissue composition and mammographic breast density in trans masculine individuals. *Breast Cancer Res*. **26**, 109 (2024). 10.1186/s13058-024-01867-w PMC11221014 38956693

[96] Di Lisa, F. S. et al. Breast and cervical cancer in transgender men: literature review and a case report. *Ther. Adv. Med. Oncol.* **16**, 17588359241259466 (2024). 10.1177/17588359241259466 PMC11316962 39131728

[97] Coelingh Bennink, H. J. T. et al. Progesterone from ovulatory menstrual cycles is an important cause of breast cancer. *Breast Cancer Res.* **25**, 60 (2023). 37254150 10.1186/s13058-023-01661-0 PMC10228093

[98] Chen, J.-H., Liu, H., Baek, H.-M., Nalcioglu, O. & Su, M.-Y. Magnetic resonance imaging features of fibrocystic change of the breast. *Magn. Reson Imaging* **26**, 1207–1214 (2008). 18436406 10.1016/j.mri.2008.02.004 PMC2613187

[99] Zhu, Y. et al. Imaging manifestations of ductal adenoma of the breast: a case report. *Open Life Sci.* **19**, 20220917 (2024). 10.1515/biol-2022-0917 PMC11252727 39022161

[100] Stachs, A., Stubert, J., Reimer, T. & Hartmann, S. Benign breast disease in women. *Dtsch. Arztebl. Int.*10.3238/arztebl.2019.0565 (2019). 10.3238/arztebl.2019.0565 PMC6794703 31554551

[101] McDonald, E. S., Clark, A. S., Tchou, J., Zhang, P. & Freedman, G. M. Clinical diagnosis and management of breast cancer. *J. Nucl. Med.* **57**, 9S–16S (2016). 26834110 10.2967/jnumed.115.157834

[102] Cardoso, F. et al. Early breast cancer: ESMO Clinical Practice Guidelines for diagnosis, treatment and follow-up†. *Ann. Oncol.* **30**, 1194–1220 (2019). 31161190 10.1093/annonc/mdz173

[103] Bleyer, A., Baines, C. & Miller, A. B. Impact of screening mammography on breast cancer mortality. *Int. J. Cancer* **138**, 2003–2012 (2016). 26562826 10.1002/ijc.29925

[104] Novack, D. V. Estrogen and bone: osteoclasts take center stage. *Cell Metab.* **6**, 254–256 (2007). 17908554 10.1016/j.cmet.2007.09.007

[105] Zhang, Y.-Y. et al. Insights and implications of sexual dimorphism in osteoporosis. *Bone Res.* **12**, 8 (2024). 38368422 10.1038/s41413-023-00306-4 PMC10874461

[106] Krugh, M. & Langaker, M. D. *Dual-Energy X-Ray Absorptiometry*. https://www.ncbi.nlm.nih.gov/books/NBK519042/ (2024). 30085584

[107] Shah, B. A., Mirchandani, A. & Abrol, S. Impact of same day screening mammogram results on women’s satisfaction and overall breast cancer screening experience: a quality improvement survey analysis. *BMC Women’s Health* **22**, 338 (2022). 35941606 10.1186/s12905-022-01919-3 PMC9361536

[108] Ghomrawi, H. M. et al. Clinicians’ perspectives on wearable sensor technology as an alternative bedside monitoring tool in two West African countries. *Int. J. Med. Inf.* **175**, 105046 (2023). 10.1016/j.ijmedinf.2023.105046 37148867

[109] Huhn, S. et al. The impact of wearable technologies in health research: scoping review. *JMIR Mhealth Uhealth* **10**, e34384 (2022). 35076409 10.2196/34384 PMC8826148

[110] Setyati, R. et al. The importance of early detection in disease management. *J. World Future Med.* **2**, 51–63 (2024).

[111] Spink, S. S. et al. High optode-density wearable diffuse optical probe for monitoring paced breathing hemodynamics in breast tissue. *J. Biomed. Optics* **26**, 062708 10.1117/1.JBO.26.6.062708 (2021). 10.1117/1.JBO.26.6.062708 PMC8170390 34080400

[112] Mahmood, S. N. et al. Full ground ultra-wideband wearable textile antenna for breast cancer and wireless body area network applications. *Micromachines* **12**, 322 (2021). 33808523 10.3390/mi12030322 PMC8003189

[113] Rahman, A., Islam, M. T., Singh, M. J., Kibria, S. & Akhtaruzzaman, M. Electromagnetic performances analysis of an ultra-wideband and flexible material antenna in microwave breast imaging: to implement a wearable medical bra. *Sci. Rep.* **6**, 38906 (2016). 28008923 10.1038/srep38906 PMC5180093

[114] Matiatou, M. et al. Complex refractive index of freshly excised human breast tissue as a marker of disease. *Lasers Med. Sci.* **37**, 2597–2604 (2022). 35301608 10.1007/s10103-022-03524-0

[115] Du, W. et al. Conformable ultrasound breast patch for deep tissue scanning and imaging. *Sci. Adv.* **9**, eadh5325 (2023). 10.1126/sciadv.adh5325 PMC10382022 37506210

[116] Pandian, R., Danasegaran, S. K., Lalithakumari, S., Rajalakshmi, G. & Kumar, G. S. Photonic crystal based hour glass patch antenna for the detection of breast cancer. *Opt. Quantum Electron* **56**, 1–11 (2024).

[117] Satpathy, S., Khalaf, O. I., Shukla, D. K., Algburi, S. & Hamam, H. Consumer electronics based smart technologies for enhanced terahertz healthcare having an integration of split learning with medical imaging. *Sci. Rep.* **14**, 1–12 (2024). 38710744 10.1038/s41598-024-58741-0 PMC11074303

[118] Elsheakh, D. M., Alsherif, S. A. & Eldamak, A. R. Textile monopole sensors for breast cancer detection. *Telecommun. Syst.* **82**, 363–379 (2023).

[119] Elsheakh, D. N., Mohamed, R. A., Fahmy, O. M., Ezzat, K. & Eldamak, A. R. Complete breast cancer detection and monitoring system by using microwave textile based antenna sensors. *Biosensors* **13**, 87 (2023). 36671922 10.3390/bios13010087 PMC9855354

[120] Vijayakumari, P. et al. Wearable transceiver with composite test-beds for breast cancer diagnosis. *Mater. Today Proc.* **45**, 3120–3123 (2021).

[121] Mukai, Y. & Suh, M. Development of a conformal woven fabric antenna for wearable breast hyperthermia. *Fash. Text.* **8**, 1–12 (2021).

[122] Elias, V., Rabih, A. & Nassar, G. Early breast lump detection using the intelligent bra”IN-bra”. *HAL Open Sci.* **4**, 43–49 (2022).

[123] Liu, L. & Webster, T. J. In situ sensor advancements for osteoporosis prevention, diagnosis, and treatment. *Curr. Osteoporos. Rep.* **14**, 386–395 (2016). 27815807 10.1007/s11914-016-0339-7

[124] Amin, U., McPartland, A., O’Sullivan, M. & Silke, C. An overview of the management of osteoporosis in the aging female population. *Women’s Health* **19**, 17455057231176655 (2023). 10.1177/17455057231176655 PMC10214060 37218715

[125] Kim, J. K., Bae, M. N., Lee, K., Kim, J. C. & Hong, S. G. Explainable artificial intelligence and wearable sensor-based gait analysis to identify patients with osteopenia and sarcopenia in daily life. *Biosensors* **12**, 167 (2022). 35323437 10.3390/bios12030167 PMC8946270

[126] Kristoffersson, A. & Lindén, M. A systematic review on the use of wearable body sensors for health monitoring: a qualitative synthesis. *Sensors* **20**, 1502 (2020). 32182907 10.3390/s20051502 PMC7085653

[127] Aggelis, D. G. et al. Fracture of human femur tissue monitored by acoustic emission sensors. *Sensors* **15**, 5803–5819 (2015). 25763648 10.3390/s150305803 PMC4435197

[128] Song, Z., Wang, B., Zhang, Z., Yu, Y. & Lin, D. A highly flexible piezoelectric ultrasonic sensor for wearable bone density testing. *Micromachines (Basel)* **14**, 1798 (2023). 37763961 10.3390/mi14091798 PMC10535184

[129] Gao, H. et al. Lower bone mineral density in patients with Parkinson’s disease: a cross-sectional study from Chinese Mainland. *Front Aging Neurosci.* **7**, 162985 (2015). 10.3389/fnagi.2015.00203 PMC4621433 26578949

[130] Stewart, A. & Black, A. Bone mineral density in osteoarthritis. *Curr. Opin. Rheumatol.* **12**, 464–467 (2000). 10990188 10.1097/00002281-200009000-00021

[131] Lodder, M. C. et al. Bone mineral density in patients with rheumatoid arthritis: relation between disease severity and low bone mineral density. *Ann. Rheum. Dis.* **63**, 1576–1580 (2004). 15547081 10.1136/ard.2003.016253 PMC1754831

[132] Redelinghuys, M. J., Geldenhuys, J., Jung, H. & Kock, M. M. Bacterial vaginosis: current diagnostic avenues and future opportunities. *Front Cell Infect. Microbiol.* **10**, 521070 (2020). 10.3389/fcimb.2020.00354 PMC7431474 32850469

[133] Eleutério, J., Campaner, A. B. & de Carvalho, N. S. Diagnosis and treatment of infectious vaginitis: proposal for a new algorithm. *Front Med. (Lausanne)* **10**, 1040072 (2023). 36844222 10.3389/fmed.2023.1040072 PMC9947655

[134] Wilson, M. & Wilson, P. J. K. Vaginitis. *Close Encounters of the Microbial Kind* 361–378 10.1007/978-3-030-56978-5_26 (2021).

[135] Rezk, S. & Alqabbasi, O. Bacterial vaginosis, vulvovaginal candidiasis, trichomonal vaginitis and aerobic vaginitis in women from Egypt. *Germs* **13**, 130 (2023). 38144250 10.18683/germs.2023.1376 PMC10746338

[136] Brown, H. & Drexler, M. Improving the diagnosis of vulvovaginitis: perspectives to align practice, guidelines, and awareness. *Popul. Health Manag.* **23**, S3–S12 (2020). 32997581 10.1089/pop.2020.0265 PMC7591372

[137] Jin, X. et al. An integrated and multi-target nucleic acid isothermal analysis system for rapid diagnosis of vulvovaginal candidiasis. *Biosensors (Basel)* **13**, 559 (2023). 37232920 10.3390/bios13050559 PMC10216317

[138] Paghi, A. et al. Wireless and flexible optoelectronic system for in situ monitoring of vaginal pH using a bioresorbable fluorescence sensor. *Adv. Mater. Technol.* **8**, 2201600 (2023).

[139] Alma - Wearable Biosensor for Monitoring Vaginal Discharge - Hackster.io. https://www.hackster.io/alma/alma-wearable-biosensor-for-monitoring-vaginal-discharge-b1022f.

[140] Pal, A., Nadiger, V. G., Goswami, D. & Martinez, R. V. Conformal, waterproof electronic decals for wireless monitoring of sweat and vaginal pH at the point-of-care. *Biosens. Bioelectron.* **160**, 112206 (2020). 10.1016/j.bios.2020.112206 32339147

[141] Torres, M. L. et al. Home-based electrochemical rapid sensor (HERS): a diagnostic tool for bacterial vaginosis. *Sensors* **23**, 1891 (2023). 36850490 10.3390/s23041891 PMC9964842

[142] Clack, K., Sallam, M., Matheson, C., Muyldermans, S. & Nguyen, N.-T. Towards a Wearable Feminine Hygiene Platform for Detection of Invasive Fungal Pathogens via Gold Nanoparticle Aggregation. *Micromachines (Basel)* **15**, 899 (2024). 10.3390/mi15070899 PMC11278863 39064410

[143] Vo, D.-K. & Trinh, K. T. L. Advances in wearable biosensors for healthcare: current trends, applications, and future perspectives. *Biosensors (Basel)* **14**, 560 (2024). 39590019 10.3390/bios14110560 PMC11592256

[144] World Health Organization. Point-of-Care Diagnostic Tests (POCTs) for Sexually Transmitted Infections (STIs). *World Health Organization* https://www.who.int/teams/sexual-and-reproductive-health-and-research-(srh)/areas-of-work/sexual-health/sexually-transmitted-infections/point-of-care-tests#:~:text=Background%20and%20rationale,professional%20and%20lay%20health%20workers.

[145] Adamson, P. C., Loeffelholz, M. J. & Klausner, J. D. Point-of-care testing for sexually transmitted infections a review of recent developments. *Arch. Pathol. Lab. Med.* **144**, 1344 (2020). 32810868 10.5858/arpa.2020-0118-RA PMC7606737

[146] Chen, L., Li, J. & Xiao, B. The role of sialidases in the pathogenesis of bacterial vaginosis and their use as a promising pharmacological target in bacterial vaginosis. *Front. Cell. Infection Microbiol.* **14**, 1367233 (2024). 10.3389/fcimb.2024.1367233 PMC10940449 38495652

[147] Wu, S. et al. A biochemiluminescent sialidase assay for diagnosis of bacterial vaginosis. *Sci. Rep.* **9**, 20024 (2019). 10.1038/s41598-019-56371-5 PMC6934538 31882933

[148] Rodríguez-Nava, C. et al. Nanophotonic sialidase immunoassay for bacterial vaginosis diagnosis. *ACS Pharm. Transl. Sci.* **4**, 365–371 (2021). 10.1021/acsptsci.0c00211 PMC7887842 33615186

[149] Ng, S. et al. Large-scale characterisation of the pregnancy vaginal microbiome and sialidase activity in a low-risk Chinese population. *NPJ Biofilms Microbiomes* **7**, 89 (2021). 10.1038/s41522-021-00261-0 PMC8688454 34930922

[150] Zhang, Y. & Rochefort, D. Fast and effective paper based sensor for self-diagnosis of bacterial vaginosis. *Anal. Chim. Acta* **800**, 87–94 (2013). 24120172 10.1016/j.aca.2013.09.032

[151] Reukov, V. et al. Fabrication of nanocoated fibers for self-diagnosis of bacterial vaginosis. *Mater. Sci. Eng. C* **29**, 669–673 (2009).

[152] Avila-Huerta, M. D. et al. Disposable device for bacterial vaginosis detection. *ACS Meas. Sci. Au.* **3**, 355–360 (2023). 37868361 10.1021/acsmeasuresciau.3c00007 PMC10588930

[153] Li, M. et al. Loop-mediated isothermal amplification (LAMP): potential point-of-care testing for vulvovaginal candidiasis. *J. Fungi* **9**, 1159 (2023). 10.3390/jof9121159 PMC10744362 38132760

[154] Mosley, G. L. et al. Improved lateral-flow immunoassays for chlamydia and immunoglobulin M by sequential rehydration of two-phase system components within a paper-based diagnostic. *Microchim. Acta* **184**, 4055–4064 (2017).

[155] Yang, J. et al. Leak-proof probe for accurate detection of Neisseria gonorrhoeae by recombinase polymerase amplification-mediated lateral flow strip. *Anal. Chim. Acta* **1258**, 341176 (2023). 10.1016/j.aca.2023.341176 37087294

[156] Ji, T. et al. Establishment and application of a rapid visual diagnostic method for Streptococcus agalactiae based on recombinase polymerase amplification and lateral flow strips. *Sci. Rep.* **14**, 10064 (2024). 10.1038/s41598-024-56138-7 PMC11066032 38698011

[157] Dighe, K. et al. Highly-specific single-stranded oligonucleotides and functional nanoprobes for clinical determination of chlamydia trachomatis and neisseria gonorrhoeae infections. *Adv. Sci.* **10**, 2304009 (2023). 10.1002/advs.202304009 PMC10754082 37870167

[158] Chen, X. et al. Nanoparticle-based lateral flow biosensor integrated with loop-mediated isothermal amplification for rapid and visual identification of chlamydia trachomatis for point-of-care use. *Front. Microbiol.* **13**, 914620 (2022). 35903464 10.3389/fmicb.2022.914620 PMC9318599

[159] Chen, X. et al. Visual and rapid identification of Chlamydia trachomatis and Neisseria gonorrhoeae using multiplex loop-mediated isothermal amplification and a gold nanoparticle-based lateral flow biosensor. *Front Cell Infect. Microbiol.* **13**, 1067554 (2023). 36926514 10.3389/fcimb.2023.1067554 PMC10011439

[160] Shivaram, K. B., Bhatt, P., Verma, M. S., Clase, K. & Simsek, H. Bacteriophage-based biosensors for detection of pathogenic microbes in wastewater. *Sci. Total Environ.* **901**, 165859 (2023). 10.1016/j.scitotenv.2023.165859 37516175

[161] Patel, D., Zhou, Y. & Ramasamy, R. P. A bacteriophage-based electrochemical biosensor for detection of methicillin-resistant staphylococcus aureus. *J. Electrochem. Soc.* **168**, 057523 (2021).

[162] Panhwar, S., Keerio, H. A., Ilhan, H., Boyacı, I. H. & Tamer, U. Principles, methods, and real-time applications of bacteriophage-based pathogen detection. *Mol. Biotechnol. 2023* 1–18 10.1007/S12033-023-00926-5 (2023). 10.1007/s12033-023-00926-5 37914863

[163] Wu, L. et al. Multiplexed detection of bacterial pathogens based on a cocktail of dual-modified phages. *Anal. Chim. Acta* **1166**, 338596 (2021). 34023003 10.1016/j.aca.2021.338596

[164] Fitzgerald, R. C., Antoniou, A. C., Fruk, L. & Rosenfeld, N. The future of early cancer detection. *Nat. Med.* **28**, 666–677 (2022). 10.1038/s41591-022-01746-x 35440720

[165] Guerra, C. E., Sharma, P. V. & Castillo, B. S. Annual Review of Medicine Multi-Cancer Early Detection: The New Frontier in Cancer Early Detection. 10.1146/annurev-med-050522 (2023). 10.1146/annurev-med-050522-033624 37729031

[166] Pink, R. C., Beaman, E. M., Samuel, P., Brooks, S. A. & Carter, D. R. F. Utilising extracellular vesicles for early cancer diagnostics: benefits, challenges and recommendations for the future. *Br J Cancer* **126**, 323–330 (2022). 10.1038/s41416-021-01668-4 PMC8810954 35013578

[167] Aziz, N. B. et al. MicroRNAs in ovarian cancer and recent advances in the development of microRNA-based biosensors. *Analyst* **145**, 2038–2057 (2020). 10.1039/c9an02263e 32016203

[168] Giampaolino, P. et al. Role of biomarkers for early detection of ovarian cancer recurrence. *Gland Surg.* **9**, 1102–1111 (2020). 10.21037/gs-20-544 PMC7475347 32953625

[169] Trinidad, C. V., Tetlow, A. L., Bantis, L. E. & Godwin, A. K. Reducing ovarian cancer mortality through early detection: approaches using circulating biomarkers. *Cancer Prev. Res.* **13**, 241–252 (2020). 10.1158/1940-6207.CAPR-19-0184 PMC7080297 32132118

[170] Lone, S. N. et al. Liquid biopsy: a step closer to transform diagnosis, prognosis and future of cancer treatments. *Mol. Cancer* **21**, 79 (2022). 10.1186/s12943-022-01543-7 PMC8932066 35303879

[171] Belotti, Y. & Lim, C. T. Microfluidics for liquid biopsies: recent advances, current challenges, and future directions. *Anal. Chem.* **93**, 4727–4738 (2021). 10.1021/acs.analchem.1c00410 33683116

[172] Lin, B. et al. Microfluidic-based exosome analysis for liquid biopsy. *Small Methods* **5**, 2001131 (2021). 10.1002/smtd.202001131 34927834

[173] Poudineh, M. et al. Tracking the dynamics of circulating tumour cell phenotypes using nanoparticle-mediated magnetic ranking. *Nat. Nanotechnol.* **12**, 274–281 (2017). 27870841 10.1038/nnano.2016.239

[174] Poudineh, M., Sargent, E. H., Pantel, K. & Kelley, S. O. Profiling circulating tumour cells and other biomarkers of invasive cancers. *Nat. Biomed. Eng.* **2**, 72–84 (2018). 10.1038/s41551-018-0190-5 31015625

[175] Gardner, L., Kostarelos, K., Mallick, P., Dive, C. & Hadjidemetriou, M. Nano-omics: nanotechnology-based multidimensional harvesting of the blood-circulating cancerome. *Nat. Rev. Clin. Oncol.* **19**, 551–561 (2022). 35739399 10.1038/s41571-022-00645-x

[176] Zhao, Z., Yang, Y., Zeng, Y. & He, M. A microfluidic ExoSearch chip for multiplexed exosome detection towards blood-based ovarian cancer diagnosis. *Lab Chip* **16**, 489–496 (2016). 26645590 10.1039/c5lc01117e PMC4729647

[177] Sinawang, P. D., Soto, F., Ozen, M. O., Akin, D. & Demirci, U. Progress and challenges in biomarker enrichment for cancer early detection. *Progr. Biomed. Eng.* **3**, 043001 (2021). 10.1088/2516-1091/ac1ea3 41367037

[178] Labib, M. et al. Tracking the expression of therapeutic protein targets in rare cells by antibody-mediated nanoparticle labelling and magnetic sorting. *Nat. Biomed. Eng.* **5**, 41–52 (2021). 32719513 10.1038/s41551-020-0590-1 PMC8436965

[179] Li, F., Yang, G., Aguilar, Z. P., Xiong, Y. & Xu, H. Affordable and simple method for separating and detecting ovarian cancer circulating tumor cells using BSA coated magnetic nanoprobes modified with folic acid. *Sens Actuators B Chem.* **262**, 611–618 (2018).

[180] Ma, J. et al. Purification of circulating tumor cells based on multiantibody-modified magnetic nanoparticles and molecular analysis toward epithelial ovarian cancer detection. *ACS Sens.* **8**, 3744–3753 (2023). 37773014 10.1021/acssensors.3c01063

[181] Gurudatt, N. G. et al. Separation detection of different circulating tumor cells in the blood using an electrochemical microfluidic channel modified with a lipid-bonded conducting polymer. *Biosens. Bioelectron.* **146**, 111746 (2019). 10.1016/j.bios.2019.111746 31586761

[182] Descamps, L., Le Roy, D. & Deman, A. L. Microfluidic-based technologies for CTC isolation: a review of 10 years of intense efforts towards liquid biopsy. *Int. J. Mol. Sci*. **23**, 1981 (2022). 10.3390/ijms23041981 PMC8875744 35216097

[183] Karabacak, N. M. et al. Microfluidic, marker-free isolation of circulating tumor cells from blood samples. *Nat. Protoc.* **9**, 694–710 (2014). 24577360 10.1038/nprot.2014.044 PMC4179254

[184] Fang, S. et al. Clinical application of a microfluidic chip for immunocapture and quantification of circulating exosomes to assist breast cancer diagnosis and molecular classification. *PLoS One* **12**, e0175050 (2017). 10.1371/journal.pone.0175050 PMC5378374 28369094

[185] Gwak, H. et al. Microfluidic chip for rapid and selective isolation of tumor-derived extracellular vesicles for early diagnosis and metastatic risk evaluation of breast cancer. *Biosens. Bioelectron.* **192**, 113495 (2021). 10.1016/j.bios.2021.113495 34273737

[186] Chen, W. et al. Simple and fast isolation of circulating exosomes with a chitosan modified shuttle flow microchip for breast cancer diagnosis. *Lab Chip* **21**, 1759–1770 (2021). 33710183 10.1039/d0lc01311k

[187] Gumus, E., Bingol, H. & Zor, E. Lateral flow assays for detection of disease biomarkers. *J. Pharm. Biomed. Anal.* **225**, 115206 (2023). 10.1016/j.jpba.2022.115206 36586382

[188] Ren, W., Mohammed, S. I., Wereley, S. & Irudayaraj, J. Magnetic focus lateral flow sensor for detection of cervical cancer biomarkers. *Anal. Chem.* **91**, 2876–2884 (2019). 30632735 10.1021/acs.analchem.8b04848

[189] Ekman, M. et al. Spectrally separated dual-label upconversion luminescence lateral flow assay for cancer-specific STn-glycosylation in CA125 and CA15-3. *Anal. Bioanal. Chem.* **13**, 3251–3260 (2024). 10.1007/s00216-024-05275-z PMC11068694 38584178

[190] Chen, J. et al. Accurate and portable tumor exosomes detection based on manganese dioxide and aptamer-functionalized fluorescent microspheres mediated dual-mode lateral flow assay. *Sens. Actuators B Chem.* **409**, 135614 (2024).

[191] Zhang, B. et al. Fluorescence quenching-based signal amplification on immunochromatography test strips for dual-mode sensing of two biomarkers of breast cancer. *Nanoscale* **9**, 18711–18722 (2017). 29165496 10.1039/c7nr06781j

[192] Guo, S. et al. All-in-one detection of breast cancer-derived exosomal miRNA on a pen-based paper chip. *Analyst* **149**, 1250–1261 (2024). 38225883 10.1039/d3an02032k

[193] Liu, H., Cao, J. & Ding, S. N. Simultaneous detection of two ovarian cancer biomarkers in human serums with biotin-enriched dendritic mesoporous silica nanoparticles-labeled multiplex lateral flow immunoassay. *Sens. Actuators B Chem.* **371**, 132597 (2022).

[194] Szymanska, B., Lukaszewski, Z., Hermanowicz-Szamatowicz, K. & Gorodkiewicz, E. A Multiple-array SPRi biosensor as a tool for detection of gynecological–oncological diseases. *Biosensors (Basel)* **13**, 279 (2023). 36832045 10.3390/bios13020279 PMC9954693

[195] Kight, E. C., Hussain, I., Bowden, A. K. & Haselton, F. R. Recurrence monitoring for ovarian cancer using a cell phone-integrated paper device to measure the ovarian cancer biomarker HE4/CRE ratio in urine. *Sci. Rep.* **11**, 21945 (2021). 34754053 10.1038/s41598-021-01544-4 PMC8578327

[196] Tellez-Gabriel, M., Knutsen, E. & Perander, M. Current status of circulating tumor cells, circulating tumor DNA, and exosomes in breast cancer liquid biopsies. *Int. J. Mol. Sci.* **21**, 1–23 (2020). 10.3390/ijms21249457 PMC7763984 33322643

[197] Jordan, N. V. et al. HER2 expression identifies dynamic functional states within circulating breast cancer cells. *Nature* **537**, 102–106 (2016). 27556950 10.1038/nature19328 PMC5161614

[198] Meng, S. et al. Surface-enhanced Raman scattering holography chip for rapid, sensitive and multiplexed detection of human breast cancer-associated MicroRNAs in clinical samples. *Biosens. Bioelectron.* 190, 113470 (2021). 10.1016/j.bios.2021.113470 34229191

[199] Yoon, J. et al. Flexible electrochemical biosensors for healthcare monitoring. *J. Mater. Chem. B* **8**, 7303–7318 (2020). 10.1039/d0tb01325k 32647855

[200] Kucherenko, I. S., Soldatkin, O. O., Kucherenko, D. Y., Soldatkina, O. V. & Dzyadevych, S. V. Advances in nanomaterial application in enzyme-based electrochemical biosensors: a review. *Nanoscale Adv.* **1**, 4560–4577 (2019). 10.1039/c9na00491b PMC9417062 36133111

[201] Tang, Y., Liu, Y., Xia, Y., Zhao, F. & Zeng, B. Simultaneous detection of ovarian cancer-concerned HE4 and CA125 markers based on Cu single-atom-triggered CdS QDs and Eu MOF@Isoluminol ECL. *Anal. Chem.* **95**, 4795–4802 (2023). 36867090 10.1021/acs.analchem.3c00273

[202] Barr, C. E., Njoku, K., Owens, G. L. & Crosbie, E. J. Urine CA125 and HE4 for the detection of ovarian cancer in symptomatic women. *Cancers (Basel)* **15**, 1256 (2023). 36831601 10.3390/cancers15041256 PMC9953976

[203] Ross, G. M. S., Filippini, D., Nielen, M. W. F. & Salentijn, G. I. J. Unraveling the Hook effect: a comprehensive study of high antigen concentration effects in sandwich lateral flow immunoassays. *Anal. Chem.* **92**, 15587–15595 (2020). 33185097 10.1021/acs.analchem.0c03740 PMC7711776

[204] Su, X. et al. Integrated SERS-vertical flow biosensor enabling multiplexed quantitative profiling of serological exosomal proteins in patients for accurate breast cancer subtyping. *ACS Nano* **17**, 4077–4088 (2023). 36758150 10.1021/acsnano.3c00449

[205] Warty, R. R., Smith, V., Patabendige, M., Fox, D. & Mol, B. Clarifying the unmet clinical need during medical device innovation in women’s health—a narrative review. *Health Care Women Int.* **45**, 1–29 (2023). 37000043 10.1080/07399332.2023.2190983

[206] Regidor, P. A., Kaczmarczyk, M., Schiweck, E., Goeckenjan-Festag, M. & Alexander, H. Identification and prediction of the fertile window with a new web-based medical device using a vaginal biosensor for measuring the circadian and circamensual core body temperature. *Gynecol. Endocrinol.* **34**, 256–260 (2018). 29082805 10.1080/09513590.2017.1390737

[207] Zhu, T. Y. et al. The accuracy of wrist skin temperature in detecting ovulation compared to basal body temperature: prospective comparative diagnostic accuracy study. *J. Med. Internet Res.* **23**, e20710 (2021). 34100763 10.2196/20710 PMC8238491

[208] Hicks, J. L. et al. Best practices for analyzing large-scale health data from wearables and smartphone apps. *npj Digit. Med.* **2**, 45 (2019). 10.1038/s41746-019-0121-1 PMC6550237 31304391

[209] Walter, J. R., Xu, S. & Rogers, J. A. From lab to life: how wearable devices can improve health equity. *Nat. Commun.* **15**, 123 (2024). 10.1038/s41467-023-44634-9 PMC10761710 38167483

[210] Mihan, A. & Van Spall, H. G. C. Interventions to enhance digital health equity in cardiovascular care. *Nat. Med.* **30**, 628–630 (2024). 10.1038/s41591-024-02815-z 38355972

[211] Dexcom. Manage your diabetes with confidence. https://www.dexcom.com/en-CA.

[212] FreeStyle. What is FreeStyle Libre? https://www.freestyle.abbott/en-ca/products/what-is-free-style-libre.html?utm_source=google&utm_medium=cpc&utm_campaign=FSL_Brand&utm_content=106128906887&utm_term=freestyle%20libre&gad_source=1&gad_campaignid=10263421839&gbraid=0AAAAADbRZUNt21gagBE8nuAAfu9mqYO2N&gclid=CjwKCAjw6NrBBhB6EiwAvnT_ruv0vdiuV0kBMFf1LsZoi-AhRfDc7RXwy5nUtJTzE-c6iMCJaZ-DGxoCNIkQAvD_BwE.

[213] Doshi, K. B., Moon, S. H., Whitaker, M. D. & Lockhart, T. E. Assessment of gait and posture characteristics using a smartphone wearable system for persons with osteoporosis with and without falls. *Sci. Rep.* **13**, 1–9 (2023). 36631544 10.1038/s41598-023-27788-w PMC9834330

[214] Huberty, J., Ehlers, D. K., Kurka, J., Ainsworth, B. & Buman, M. Feasibility of three wearable sensors for 24 hour monitoring in middle-aged women. *BMC Women’s Health* **15**, 1–9 (2015). 26223521 10.1186/s12905-015-0212-3 PMC4518514

[215] Sanchez-Trigo, H., Maher, C., Godino, J. G. & Sañudo, B. Effects of an mHealth physical activity intervention to prevent osteoporosis in premenopausal women. A randomized controlled trial. *J. Sci. Med. Sport* **26**, 545–552 (2023). 37739855 10.1016/j.jsams.2023.09.004

[216] Sánchez-Trigo, H., Sanchez-Oliver, A. J., Abt, G. & Sañudo, B. Validation of a Wearable accelerometer-based activity monitor for use in future osteoporosis prevention programs. *Sustainability* **12**, 2187 (2020).

[217] Navalta, J. W., Ramirez, G. G., Maxwell, C., Radzak, K. N. & McGinnis, G. R. Validity and reliability of three commercially available smart sports bras during treadmill walking and running. *Sci. Rep.* **10**, 1–9 (2020). 32355249 10.1038/s41598-020-64185-z PMC7192924

[218] Arcarisi, L. et al. Palpreast—a new wearable device for breast self-examination. *Appl. Sci.* **9**, 381 (2019).

[219] Park, C. K. S. et al. Cost-effective, portable, patient-dedicated three-dimensional automated breast ultrasound for point-of-care breast cancer screening. *Sci. Rep.* **13**, 1–16 (2023). 37658125 10.1038/s41598-023-41424-7 PMC10474273

[220] Broach, R. B. et al. A cost-effective handheld breast scanner for use in low-resource environments: a validation study. *World J. Surg. Oncol.* **14**, 1–6 (2016). 27793162 10.1186/s12957-016-1022-2 PMC5084434

[221] Fertility Test Market by Product (Ovulation Predictor Kits, Fertility Monitors (Urine, Saliva, Blood)), Mode of Purchase (OTC, Prescription, Online), Application (Female, Male), End User (Home care, Fertility clinics, hospitals) & Region - Global Forecast to 2025. https://www.marketsandmarkets.com/Market-Reports/fertility-testing-devices-market-139945432.html#:~:text=Market%20Growth%20Outlook%20Summary,both%20developed%20and%20developing%20countries.

[222] Gestational Diabetes Market Size And Share Analysis - Growth Trends And Forecasts (2025-2032). https://www.coherentmi.com/industry-reports/gestational-diabetes-market.

[223] Patenting products for women. https://www.taylorwessing.com/en/insights-and-events/insights/2024/06/patenting-products-for-women.

[224] Mshelia, Y. U., Elei, F. O., Ikerionwu, C. O., Erike, A. I. & Mshelia, Y. U. Transition to Value-Based Care IoT Wearable Devices in Maternity Healthcare for Low-Resource Countries. *Int. J. Sci. Res. Comput. Sci. Eng.* **11**, 8–14 (2023).

[225] Godfrey, A. et al. Wearables beyond borders: a case study of barriers to gait assessment in low-resource settings. *Maturitas* **137**, 7–10 (2020). 32498939 10.1016/j.maturitas.2020.04.013

[226] Paolillo, E. W. et al. Wearable use in an observational study among older adults: adherence, feasibility, and effects of clinicodemographic factors. *Front Digit Health* **4**, 884208 (2022). 35754462 10.3389/fdgth.2022.884208 PMC9231611

[227] Li, J., Ma, Q., Chan, A. H. & Man, S. S. Health monitoring through wearable technologies for older adults: smart wearables acceptance model. *Appl. Erg.* **75**, 162–169 (2019). 10.1016/j.apergo.2018.10.006 30509522

[228] Tikkanen, H., Heinonen, K. & Ravald, A. Smart wearable technologies as resources for consumer agency in well-being. *J. Interact. Mark.* **58**, 136–150 (2023).

[229] Yao, R. et al. Inequities in health care services caused by the adoption of digital health technologies: scoping review. *J. Med. Internet Res.* **24**, e34144 (2022). 35311682 10.2196/34144 PMC8981004

[230] Biga, R., Nottebaum, S., Kozlakidis, Z. & Psomiadis, S. Digitalization of Healthcare in LMICs: Digital Health and the Digital Divide Based on Technological Availability and Development. In 185–193. 10.1007/978-3-031-62332-5_18 (2024).

[231] Borges do Nascimento, I. J. et al. Transforming women’s health, empowerment, and gender equality with digital health: evidence-based policy and practice. *Lancet Digit. Health*10.1016/j.landig.2025.01.014 (2025). 10.1016/j.landig.2025.01.014 PMC12208921 40368744

[232] Berrian, K., Exsted, M. D., Lampe, N. M., Pease, S. L. & Akré, E. L. Barriers to quality healthcare among transgender and gender nonconforming adults. *Health Serv. Res.* **60**, e14362 (2025). 10.1111/1475-6773.14362 PMC11782051 38988141

[233] Renner, J. et al. Barriers to accessing health care in rural regions by transgender, non-binary, and gender diverse people: a case-based scoping review. *Front. Endocrinol. (Lausanne)* **12**, 717821 (2021). 10.3389/fendo.2021.717821 PMC8637736 34867775

[234] Safer, J. D. & Chan, K. J. Review of medical, socioeconomic, and systemic barriers to transgender care. In 25–38. 10.1007/978-3-030-05683-4_2 (2019).

[235] Cook, R. Women’s Health and Human Rights The Promotion and Protection of Women’s Health through International Human Rights law. *World Health Organization Geneva.* https://iris.who.int/bitstream/handle/10665/39354/9241561661_eng.pdf;jsessionid=314C6EB3C086C (1994).

[236] Women’s autonomy, equality and reproductive health | OHCHR. https://www.ohchr.org/en/special-procedures/wg-women-and-girls/womens-autonomy-equality-and-reproductive-health.

[237] 2024 – Women’s Health Manifesto – Eurohealth. https://eurohealth.ie/2023/05/29/2024-womens_health_manifesto/.

[238] Daniel, H. et al. Women’s health policy in the united states: an american college of physicians position paper. *Ann. Intern Med.* **168**, 874–875 (2018). 29809243 10.7326/M17-3344

[239] Women’s health and well-being in Europe: beyond the mortality advantage. https://www.who.int/europe/publications/i/item/9789289051910 (2016).

[240] Women’s Health Initiative (WHI) | NHLBI, NIH. https://www.nhlbi.nih.gov/science/womens-health-initiative-whi.

[241] National Women’s Health Strategy (2020-2030) - Australian Women’s Health Alliance. https://australianwomenshealth.org/resource/national-womens-health-strategy/.

[242] United Nations. United Nations Population Fund. *United Nations*. https://www.unfpa.org/donate/EmergencyBirthKit/b?utm_source=google&utm_medium=cpc&utm_campaign=UNFPA_DLV_GAdsP_Search_Brand_Alpha_Tier1_EN&utm_content=Evergreen&gad_source=1&gad_campaignid=22480363807&gbraid=0AAAAAoaU5jLKIgEPYbRPuycqhN2wHuvDE&gclid=EAIaIQobChMI3L35tt26jwMVW1N_AB3W6TrWEAAYASAAEgKmavD_BwE.

[243] World Health Organization. Global Strategy for Women’s, Children’s and Adolescents’ Health Data Portal. *World Health Organization*. https://platform.who.int/data/maternal-newborn-child-adolescent-ageing/global-strategy-data.

[244] Ye, J., Hai, J., Song, J. & Wang, Z. The role of artificial intelligence in the application of the integrated electronic health records and patient-generated health data. *medRxiv* 2024.05.01.24306690 10.1101/2024.05.01.24306690 (2024).

[245] Wang, Z. et al. AI-assisted diagnosis of vulvovaginal candidiasis using cascaded neural networks. *Microbiol. Spectr.* **13**, e01691–24 (2025). 10.1128/spectrum.01691-24 PMC11705804 39576103

[246] Mennickent, D. et al. Machine learning applied in maternal and fetal health: a narrative review focused on pregnancy diseases and complications. *Front. Endocrinol. (Lausanne)* **14**, 1130139 (2023). 10.3389/fendo.2023.1130139 PMC10235786 37274341

[247] Zhang, Z. et al. Machine learning prediction models for gestational diabetes mellitus: meta-analysis. *J. Med. Internet Res.* **24**, e26634 (2022). 35294369 10.2196/26634 PMC8968560

[248] Foschi, C. et al. Potential use of artificial intelligence for vaginal swab analysis in the assessment of common genital disorders: a pilot study. *New Microbiol.* **45**, 291 (2022). 36190372

[249] Zhu, X. et al. Cervical cancer screening aided by artificial intelligence, China. *Bull. World Health Organ.* **101**, 381 (2023). 37265676 10.2471/BLT.22.289061 PMC10225939

[250] McKinney, S. M. et al. International evaluation of an AI system for breast cancer screening. *Nature* **577**, 89–94 (2020). 31894144 10.1038/s41586-019-1799-6

[251] Ng, A. Y. et al. Prospective implementation of AI-assisted screen reading to improve early detection of breast cancer. *Nat. Med.* **29**, 3044–3049 (2023). 37973948 10.1038/s41591-023-02625-9 PMC10719086

[252] Salim, M. et al. AI-based selection of individuals for supplemental MRI in population-based breast cancer screening: the randomized ScreenTrustMRI trial. *Nat. Med.*10.1038/s41591-024-03093-5 (2024). 10.1038/s41591-024-03093-5 PMC11405258 38977914

[253] van Dooijeweert, C. et al. Clinical implementation of artificial-intelligence-assisted detection of breast cancer metastases in sentinel lymph nodes: the CONFIDENT-B single-center, non-randomized clinical trial. *Nat. Cancer* **5**, 1195–1205 (2024). 38937624 10.1038/s43018-024-00788-z PMC11358151

[254] Lotter, W. et al. Robust breast cancer detection in mammography and digital breast tomosynthesis using an annotation-efficient deep learning approach. *Nat. Med.* **27**, 244–249 (2021). 33432172 10.1038/s41591-020-01174-9 PMC9426656

[255] Burks, J. H. et al. General feature selection technique supporting sex-debiasing in chronic illness algorithms validated using wearable device data. *npj Women’s Health* **2**, 37 (2024).

[256] Simms, K. T. et al. Benefits, harms and cost-effectiveness of cervical screening, triage and treatment strategies for women in the general population. *Nat. Med.* **29**, 3050–3058 (2023). 38087115 10.1038/s41591-023-02600-4 PMC10719104

[257] Endo, P. T. Artificial intelligence for women and child healthcare: is AI able to change the beginning of a new story? A perspective. *Health Sci. Rep.* **8**, e70779 (2025). 10.1002/hsr2.70779 PMC12053047 40330751

[258] Massachusetts General Hospital. Are you pregnant? Do you own a smartphone? *MGH’s Center for Women’s Mental Health*. https://rally.massgeneralbrigham.org/study/mghpdsmobileapp (2025).

[259] Pearlstein, T., Howard, M., Salisbury, A. & Zlotnick, C. Postpartum depression. *Am. J. Obstet. Gynecol.* **200**, 357–364 (2009). 19318144 10.1016/j.ajog.2008.11.033 PMC3918890

[260] Davidson, L. & Boland, M. R. Towards deep phenotyping pregnancy: a systematic review on artificial intelligence and machine learning methods to improve pregnancy outcomes. *Brief Bioinform.* **22**, bbaa369 (2021). 10.1093/bib/bbaa369 PMC8424395 33406530

[261] OvuFirst Wearable Fertility Tracker. https://www.hellovio.com/en_ca/about-ovufirst/.

[262] GE HealthCare (United States). Invenia Abus Breast Imaging Ultrasound. https://www.gehealthcare.com/products/ultrasound/breast-ultrasound/invenia-abus.

[263] Futoma, J., Simons, M., Panch, T., Doshi-Velez, F. & Celi, L. A. The myth of generalisability in clinical research and machine learning in health care. *Lancet Digit Health* **2**, e489–e492 (2020). 32864600 10.1016/S2589-7500(20)30186-2 PMC7444947

[264] Achtari, M. et al. Gender bias in AI’s perception of cardiovascular risk. *J. Med. Internet Res.* **26**, e54242 (2024). 39437384 10.2196/54242 PMC11538872

[265] Bruce, L. K. et al. Variability of temperature measurements recorded by a wearable device by biological sex. *Biol. Sex. Differ.* **14**, 76 (2023). 37915069 10.1186/s13293-023-00558-z PMC10619297

[266] Smarr, B. L. AI for precision medicine must keep non-random complexity in mind to support equity in outcomes. In *2024 IEEE 20th International Conference on e-Science (e-Science)* 1–7 (IEEE, 2024). 10.1109/e-Science62913.2024.10678664.

[267] Cirillo, D. et al. Sex and gender differences and biases in artificial intelligence for biomedicine and healthcare. *NPJ Digit Med.* **3**, 81 (2020). 32529043 10.1038/s41746-020-0288-5 PMC7264169

[268] Murrin, E. M., Saad, A. F., Sullivan, S., Millo, Y. & Miodovnik, M. Innovations in diabetes management for pregnant women: artificial intelligence and the internet of medical things. *Am. J. Perinatol.*10.1055/a-2489-4462 (2024). 10.1055/a-2489-4462 39592107

[269] Abu-El-Ruz, R. et al. Artificial intelligence in bacterial infections control: a scoping review. *Antibiotics* **14**, 256 (2025). 40149067 10.3390/antibiotics14030256 PMC11939793

