# Supplementary material for: Advances in biomonitoring technologies for women’s health
Source: Nat Commun. 2025 Sep 26;16:8507. doi: 10.1038/s41467-025-63501-3 (PMC12475061; doi:10.1038/s41467-025-63501-3)
Supplement: Supplementary file 1 — Supplementary Information [file 41467_2025_63501_MOESM1_ESM.pdf]

Supplementary information

Advances in Biomonitoring Technologies for Women's Health

Shaghayegh Moghimikandelousi[1,8], Lubna Najm[2,8], Yerim Lee[3,8], Fereshteh Bayat[2], Akansha Prasad[1], Shadman Khan[3], Aishwarya Bhavan[4], Wei Gao[3*], Zeinab Hosseinidoust[1,5,6,7*], Tohid F. Didar[1,2,7*]

[1]School of Biomedical Engineering, McMaster University, 1280 Main Street West, Hamilton, ON, Canada.

[2]Department of Mechanical Engineering, McMaster University, 1280 Main Street West, Hamilton, ON, Canada.

[3]Andrew and Peggy Cherng Department of Medical Engineering, Division of Engineering and Applied Science, California Institute of Technology, Pasadena, CA, USA.

[4]Department of Biochemistry and Biomedical Sciences, McMaster University, 1280 Main Street West, Hamilton, ON, Canada

[5]Department of Chemical Engineering, McMaster University, 1280 Main Street West, Hamilton, ON, Canada.

[6]Farncombe Family Digestive Health Research Institute, McMaster University, Hamilton, Ontario, Canada.

[7]Michael DeGroote Institute for Infectious Disease Research, McMaster University, Hamilton, Ontario, Canada.

[8]These authors contributed equally to this work.

*e-mail: weigao@caltech.edu; doust@mcmaster.ca; didart@mcmaster.ca

Supplementary Table 1. Wearable technologies for women's health biomonitoring: modalities, materials and applications.

Wearable	Sensing modality	Material	Test group	Testing strategy	End user application	Ref.
**Wearable technologies for biometric monitoring**						
**Biocompatible intravaginal logger**	Temperature	Silicone elastomer with casing made of epoxy and alumina	*In vivo* cynomolgus macaques' model	Measures intravaginal temperature continuously	Utilized for everyday continuous, long-term tracking of period cycles after use of contraception	1
**Automated pulse rate nocturnal bracelet**	Pulse rate sensing	photoplethysmography (PPG) sensors embedded in bracelet	91 female participants	Measures wrist pulse with real-time continuous data collection	Automated pulse monitoring bracelet, worn regularly at night to determine fertility windows	2
**Conductive textile bodysuit with integrated ECG**	ECG	Cotton and lycraTM	5 pregnant female participants	Using abdominal ECG of fetal and maternal heartrate for wireless pregnancy monitoring	Elastic flexible bodysuit with smart textile for telemedicine remote monitoring of pregnancy	3
**Soft sensor wearable biometrics system**	ECG, EHG, doppler ultrasound, thermal, PPG, heart rate	Gold and metal electrodes encased in silicone housing with hydrogel adhesive used for placement on skin and signal transfer	576 pregnant female participants	Measures heart rate, ultrasound, pulse, cardiac health, uterine contraction, oxygenation of capillaries and peripheral temperature	Utilized for real-time, all-encompassing monitoring of fetal and maternal during late stages of pregnancy and labor	4
**Wearable technologies for hormone monitoring**						
**Conductive yarn Wearable (Belly Band)**	Radio frequency identification (RFID) strain and bio signal sensor	Conductive yarn and flexible PCB components	Pregnancy Simulation	Using abdominal bio signals and strain measurements for wireless pregnancy monitoring	Flexible yarn worn over the belly, sensing thrombosis, heart rate, and uterine contractions for pregnancy monitoring	5
**Automated nano biosensor, hydrogel microfluidic wearable patch**	Multiplexed electrochemical detection of oestradiol and oH	Gold nanoparticle-Mxene on carbon electrode	Female participants	Collect sweat samples through iontophoresis hydrogels and microfluidics, which are tested in multiplex nano biosensor array	Automated sweat sample collection using hydrogel microfluidic platform, followed by multiplex detection for accurate fertility monitoring	6

**Cortisol detecting carbon yarn for smart textile wearables**	Electrochemical detection of cortisol	Fe_2O_3 and Carbon composite yarn functionalized with EDC/NHS	*In vitro* detecting in sweat samples	Integrated into clothing and detects cortisol levels from sweat collected by monoclonal antibody functionalized carbon yarn	Detecting cortisol to determine bone mineral density, allowing for real time osteoporosis detection in smart fabric wearables	7
**Cortisol detecting carbon yarn for smart textile wearables**	Electrochemical detection of cortisol	Fe_2O_3 and Carbon composite yarn functionalized with EDC/NHS	*In vitro* detecting in sweat samples	Integrated into clothing and detects cortisol levels from sweat collected by	Detecting cortisol to determine bone mineral density, allowing for real time	7

				monoclonal antibody functionalized carbon yarn	osteoporosis detection in smart fabric wearables	
**Wearable technologies for Imaging**						
**Acoustic emission wearable fracture sensors**	Acoustic wave induction and acquisition	Fabric	*in situ* 11 human femurs from cadavers	Measures acoustic wave peaks and rise times in pico range to detect start of fractures	Monitors osteoporotic bone for risk of bone fracture at different types of loads	8
**In-sole gait wearables**	IMU sensing	In-sole plastic and woven materials	42 female participants (over 65 years old), half with osteopenia and half without osteopenia	Measures IMU spatial-temporal and kinetics parameters for wireless monitoring	Detects gait parameters to determine risk of Parkinson's Disease, fall risk, total hip arthroplasty and sarcopenia in females with osteopenia	9
**Ultrasonic piezoelectric wearable band**	Axial transmission ultrasonic wave transmission and acquisition	PMN-PZT piezoelectric ceramic on PDMS substrate	1 healthy, 25-year-old male participant	Measures response voltage of axial transmitted and received ultrasonic wave	Bone density testing and imaging for real-time bone monitoring and osteoporosis detection	10
**Bioimpedance electrode system for wearables applications**	Injection module inducing current (1kHz, 5V, 0.9mA), with acquisition module of two impedance electrodes	Metal	40 female participants	Bioimpedance and electrical current conductivity monitoring within malignant breast tissue	Sense changes in impendence of induced current within breast to determine malignant vs benign tissues for early-onset diagnostics	11
**Omnidirectional bistatic radar-microwave imaging**	Microwave impedance induction and acquisition model, in single probe	5-(4-(perfluorohexyl) phenyl) thiophene-2-carbaldehyde biodegradable compound	Tumor simulating phantom	Measuring reflection and backscattering of microwaves for tumor imaging	Senses microwave reflection for tumor imaging for applications in one time use monitoring smart bra	12
**Bio-inspired honeycomb clinical breast patch containing piezoelectric crystals**	Ultrasound detection and imaging	Piezoelectric crystal [Yb/Bi-Pb(In1/2Nb1/2)O3-Pb(Mg1/3Nb2/3) O3-PbTiO3] (Yb/Bi-PIN-PMN-PT)	Female participant	Measures ultrasound wave frequencies	Leverages ultrasound technology for deep tissue modelling of breasts for one-time clinical diagnostic use	13
**Heat-reduction mastectomy bra**	8 humidity and temperature dual modality sensors	Nylon, polyester, spandex, polyurethane	9 healthy male participants, wearing mastectomy prosthesis	Dual modality sensors were built into bra materials: 4 placed on skin, 4 placed on prosthesis	Sense temperature and humidity during physical activity, allowing for heat dissipation and increased comfort wearing bra for women after mastectomy	14
**Woven textile temperature sensors for wearables applications**	Temperature sensor and induction systems	Copper plated polyester	Tumor simulating phantom	Measuring and inducing temperature in breast tissue	Sense temperature to provide hyperthermia therapy to breast tissues	15

**Wide dual-band textile antenna microstrips**	Microwave impedance induction and acquisition model, in single probe	Epoxy, cotton, polyester	Tumor simulating phantom	Measures microwave frequencies	Leverages microwave imaging technology and devices for comprehensive breast imaging, with existing textile fabrication methods	16
**Circularly polarized textile antennas**	Microwave induction and acquisition	Cotton, polyester, denim, crepe	Tumor simulating breast phantom	Measures bandwidth and specific absorption rate of 1.8–8 GHz frequency microwaves	Measures microwave absorption in circularly polarized, compared to traditional linearly polarized, for integration in smart bra	17
**Cancer cell hydrogel detection with self-healing properties**	Electrochemical glutathione/ROS detection with wireless read out	Ureidopyriminone-conjugated gelatin (Gel-UPy) embedded with diselenide-containing carbon dots (dsCD)	*In vivo* tumor mouse model	Measuring cleavage of diselenide bonds from glutathione/ROS and inducing formation of hydrogen bonds	Cancer cell monitoring and induction of tumor self-healing inflammatory responses, worn as an adhesive patch on skin	18
**Nanoparticle embedded wearable films**	Impedance and dielectric strain induction and acquisition model	Thermoplastic polyurethane embedded with hafnium oxide nanoparticles (HfO2 NPs)	*In vivo* tumor mouse model	Measures film deformation and electrical current strain/conductivity	Monitoring tumor volume for real-time continuous tumor growth/regression detection, followed by informing and induction of sonodynamic therapy	19
**NIR and MSOT nanoprobe**	Fluorescence signals and absorption	Nitrobenzyloxydiphenylamino and quinolinium	*In vivo* tumor mouse model	Measures levels of nitroreductase	Nanoprobe fluorescence monitoring of metastasis in NIR-I/II and MSOT imaging, to inform and induce cancer treatment	20

Supplementary Table 2. Diagnostic technologies for women's cancer detection.

Technology	Biomarkers	Cancer type	Sample	Sensitivity	Summary of method	Ref
**Multiplex detection**	Claudin 7, CD81	Breast	Plasma	Claudin7=0.4 pg ml^{-1}<br>CD81= 3 pg ml^{-1}	A paper-based microfluidic sandwich-like electrochemical biosensor detects breast cancer extracellular vesicles using two different antibody-modified channels and HRP enzyme activity.	21
**Multiplex detection**	CA125, carcinoembryonic antigen (CEA) serum alpha-fetoprotein (AFP)	Ovarian	Serum	AFP=30 ng ml^{-1}<br>CEA=50 ng ml^{-1}<br>CA125=80 U ml^{-1}	Sandwich-like suspension arrays immunoassay based on Photonic crystal (PhC) barcodes. Antibody-modified dendritic silica nanoparticles ($dSiO_2$) and antibody-modified CdTe QDs used as detector.	22
**Multiplex detection/LFAs**	HPV16, HPV18, and HPV45	Cervical	Cervicovaginal swab samples	HPV16=100 copies/reaction<br>HPV18=10copies/reaction<br>HPV45=10copies/reaction	Loop mediate isothermal amplification-based LFA.<br>The focus of this paper was on amplification of DNA.	23
**Multiplex detection/LFAs**	CEA, carbohydrate antigen (CA153)	Breast	Serum	CEA=0.06 ng ml^{-1}<br>CA153=0.09 U ml^{-1}	Dual mode detection (florescent and colorimetric) LFA. Samples preconcentrated with Cy5-antibody-modified PLGA@Fe_3O_4 super-paramagnetic nanosphere then detected with antibody-modified strip.	24
**Multiplex detection/LFAs**	Exosomal proteins, HER2 and MUC1	Breast	Serum	10^6 particles ml^{-1} for both exosomes.	Surface-enhanced Raman spectroscopy (SERS)-based LFA that is modified with capture aptamer.	25

Supplementary Table 3. Commercial wearables in women's health: applications and market status.

Commercial Product	Company	Patent Scope	Women Health Application	Commercial Status	Ref
**Commercial products for women's cancer biomarker isolation and screening**					
**CELLSEARCH® circulating tumor cell test**	Menarini Silicon Biosystems, Inc	WO2010052543A1, US20190293632A1, US10234447B2, KR101680619B1, EP2350647B1, JP5814124B2, ES2910715T3, EP3185012B1, CA2742769C, CN102272595B	Isolates and enumerates from patient blood samples for cancer diagnostics	Completed pilot studies, received FDA approval and are at initial stages of market introduction	26
**Commercial products for detecting bacterial, parasitic and yeast vaginal infections**					
**OSOM® BVBLUE® Test**	SEKISUI Diagnostics, LLC	WO2013119729A1, US9297724B2	Colorimetrically detects SLD levels in vaginal fluids for diagnosing bacterial vaginosis	Available for consumer purchase in Africa, Europe, Latin America, Middle East, and the USA	27
**Vagisil® screening kit**	Combe Inc	No available patents	Colorimetrically detects vaginal pH of 5 from swabbed samples, indicative of bacterial vaginal infections	Available for consumer purchase	28
**CVS health feminine screening kit for vaginal health**	CVS Health	No available patents	Colorimetrically detects abnormal vaginal pH from swabbed samples, indicative of vaginal yeast infections	Available for consumer purchase	29
**AZO® vaginal pH test**	i-Health, Inc	CN204302188U	Colorimetrically detects vaginal pH of 5 from swabbed samples, indicative of vaginal bacterial or yeast infections	Available for consumer purchase	30
**VagiSense®**	Paladin Pharma' Consumer Healthcare	No available patents	Colorimetrically detects abnormal vaginal pH from swabbed samples, indicative of vaginal bacterial, parasitic or yeast infections	Available for consumer purchase	31
Commercial products for wearable biomonitoring of women's health					
**Cyrcadia breast monitor**	Cyrcadia Health	US8185485B2	Measures circadian temperature changes in breast cells using smart patches and a smartphone app	Currently conducting pilot study with 173 breast cancer patients	32
**iBreastExam®**	UE LifeSciences Inc	No available patents	Measures tissue stiffness for detecting tumor abnormalities as a handheld device with smart phone app	FDA approved and available for clinicians in 12 countries	33
**Invenia ABUS 2.0,**	GE HealthCare	US20150087979A1	Images breast tissue for cancer lesion detection using handheld device and AI assistant	FDA approved and available for clinicians in the USA	34
**Sensoria Smart Bra**	Sensoria Fitness	WO2017185050A1, US20190094088A1	Measures heart rate biometrics	Available for purchase by consumers	35
**SmartBra EZ Rose®**	IcosaMed Sàrl	Patent pending	Uses ultrasound transmissions to detect breast tissue density, identifying stiffness indicative of cancer through wireless data transfer	Completed initial product testing, and currently conducting scale up for market introduction	36

**Palpreast**	UBORA	No available patents	Uses pressure sensing textiles to detect superficial breast tumors, lesions and stiff tissues	In development and initial testing	[37]
**Ava Bracelet**	Ava, Inc	WO2016131630A1, EP3258853B1, CN107278139B, US10779802B2	Measures temperature, accelerometry and resting pulse rate at night to monitor fertility cycles and pregnancy	Completed clinical trials, received FDA approval and available on the market	[38]
**OvuFirst**	viO HealthTech Inc	US20050192512A1, US8496592B2, US9155522B2, AU2007293287B2, ES2366929T3, CN101528138B, JP2010502338A, WO2008029130A2	Measures temperature during sleep for monitoring fertility cycles using wireless OvuSense App and AI Assistant	Available for purchase by consumers	[39]
**OvulaRing**	VivoSensMedical GmbH	EP2567680B1, EP3048984B1, EP3975871A1, US9314227B2, US10321897B2, US20220218314A1, CN113873953A, WO2015044398A1	Measures core body temperature throughout the day using a intravaginal biosensor, with wireless smartphone app and AI Assistant	Available for purchase by consumers	[40]
**kegg™**	Lady Technologies Inc	WO2019118985A1, US20200297327A1, USD896377S1, US20220071603A1, EP3749217A4	Measures electrolyte levels in cervical fluids for rapid fertility monitoring	Received FDA approval and available for purchase by consumers	[41]
**TempDrop™**	Tempdrop Inc.	USD746161S1	Measures BBT during sleep to monitor fertility windows with wireless smartphone app	Received FDA approval and available for purchase by consumers	[42]
**OuraRing**	Ōura Health	WO2015081321A1, US11868179B2, US9582034B2, US10126779B2, US10331168B2, US11868179B2, US11874702B2, CN106104408B, EP4071581A1, JP2017506376A, CA2931973A1,	Measures multiple biometrics to monitor fertility, menstrual health and pregnancy with wireless smartphone app	Received FDA approval and CE certification, available for consumer purchase	[43]
**EmbracePlus, E4 Wristband**	Empatica Inc.	USD867599S1	Measures multiple biometrics to monitor fertility, menstrual health and pregnancy with wireless smartphone app	Received FDA approval and CE certification, available for clinicians and researcher purchase	[44]
**femSense**	SteadySense GmbH	WO2020053317A1, EP3849402C0, US11986272B2, ES2959843T3, CA192065S, CA192066S	Measures basal body temperature using a waterproof sensor patch to monitor fertility windows with wireless smartphone app	Received FDA approval and available for purchase by consumers	[45]
**Nuvo**	NUVO Inc	WO2016142780A1, US9392952B1, US10111600B2, CA2979135C, AU2016230825B2, CN107405096B, KR101900641B1,	Measures maternal heart rate, fetal heart rate and uterine contractions using sensing waist band with remote telemedicine communications during pregnancy	Received FDA approval and available for clinicians to prescribe to pregnant patients	[46]

		EP3267884B1, ES2895642T3,			
**Bloomlife Connects**	Bloomlife	No patents available	Measures biometrics of pregnancy using operating system downloadable on certified monitoring devices	Available for consumer purchase	[47]
**Muvone**	Secmotic	No patents available	Measures accelerometry and motion sensor to monitor physical activity to prevent osteoporosis development	Currently conducting market introduction testing	[48]
**ActiGraph wGT3X-BT®**	ActiGraph Inc	Patent pending	Measures motion and gait patterns using wristband with remote telemedicine communications	Received FDA approval and available for purchase by clinicians and researchers	[49]
**MoveMonitor**	McRoberts	Patent pending	Measures motion and accelerometry using wearable sensor with remote telemedicine communications for gait monitoring applications	Received FDA approval and CE certification, conducting initial market testing amongst researchers and clinicians	[50]
**OsteoBoost**	Bone health technologies	WO2014099527A1, JP7186691B2, KR102630817B1, US10206802B2, US11026824B2, US11219542B2, US11806262B2, US20240024142A1	Measures bone density with feedback vibration system to improve bone health with wearable waist band	Currently conducting clinical trials and patient testing	[51]

Supplementary Table 4. AI algorithm for women's health applications.

Disease	Algorithm	Data sets	Accuracy/ area under receiver operating characteristic curve (AUC)	References
**GDM**	Mutual Information (MI)	Qatari population first-trimester clinical data	88.8%	52
**GDM**	Model Risk of Bias Assessment Tool (PROBAST)	The PubMed, Web of Science, IEEE Xplore, and China National Knowledge Infrastructure databases	84.94%	53
**GDM**	LR	4771 pregnant women in early gestation in Xinhua Hospital	AUC of 0.766	54
**Preeclampsia**	The gradient boosting	All births (n=16,370) at Lucile Packard children hospital at Stanford, California	AUC of 0.89	55
**Preeclampsia**	Tree model<br>naïve Bayes classification<br>Support vector machine<br>random forest algorithm<br>stochastic gradient boosting method<br>LR	11,006 pregnant women who received antenatal care at Yonsei University Healthcare Center in Seoul, Korea	AUC of 0.857<br>0.776<br>0.573<br>0.894<br>0.924<br>0.806	56
**Pregnancy-induced hypertensive**	ANN<br>Multi logistic variant regression	303 consecutive normotensive white women at high risk for PE and IUGR, who attended the prenatal medicine unit of the institute of obstetrics and gynecology of the university of Florence for preconception counselling.	AUC of 0.952<br>0.962	57
**Still birth**	Two-step stacked ensemble (SE) classifier	Iranian Maternal and neonatal registry dataset is used in this study considering all births at or beyond 28th gestational week	90%	58
**Still birth**	LR<br>Decision tree<br>random forest algorithm<br>XGBoost<br>Multi-layer Perceptron	The data linkage branch (DLB) of the WA department of health,	AUC from 0.52 to 0.84	59
**VVC**	YOLO model	123 vaginal discharge slides from patients treated at Changsha Hospital for Maternal & Child Health Care	93%	60

References

1. Boyd, P. *et al.* A Temperature-Monitoring Vaginal Ring for Measuring Adherence. *PLoS One* 10, e0125682 (2015).

2. Shilaih, M., Clerck, V. De, Falco, L., Kübler, F. & Leeners, B. Pulse Rate Measurement During Sleep Using Wearable Sensors, and its Correlation with the Menstrual Cycle Phases, A Prospective Observational Study. *Scientific Reports 2017 7:1* 7, 1–7 (2017).

3. Signorini, M. G., Lanzola, G., Torti, E., Fanelli, A. & Magenes, G. Antepartum Fetal Monitoring through a Wearable System and a Mobile Application. *Technologies 2018, Vol. 6, Page 44* 6, 44 (2018).

4. Ryu, D. *et al.* Comprehensive pregnancy monitoring with a network of wireless, soft, and flexible sensors in high-and low-resource health settings. *Proc Natl Acad Sci U S A* 118, e2100466118 (2021).

5. Mongan, W. *et al.* A Multi-Disciplinary Framework for Continuous Biomedical Monitoring Using Low-Power Passive RFID-Based Wireless Wearable Sensors. *2016 IEEE International Conference on Smart Computing, SMARTCOMP 2016* (2016) doi:10.1109/SMARTCOMP.2016.7501674.

6. Ye, C. *et al.* A wearable aptamer nanobiosensor for non-invasive female hormone monitoring. *Nature Nanotechnology 2023 19:3* 19, 330–337 (2023).

7. Sekar, M., Pandiaraj, M., Bhansali, S., Ponpandian, N. & Viswanathan, C. Carbon fiber based electrochemical sensor for sweat cortisol measurement. *Scientific Reports 2019 9:1* 9, 1–14 (2019).

8. Aggelis, D. G. *et al.* Fracture of Human Femur Tissue Monitored by Acoustic Emission Sensors. *Sensors 2015, Vol. 15, Pages 5803-5819* 15, 5803–5819 (2015).

9. Kim, J. K., Bae, M. N., Lee, K., Kim, J. C. & Hong, S. G. Explainable Artificial Intelligence and Wearable Sensor-Based Gait Analysis to Identify Patients with Osteopenia and Sarcopenia in Daily Life. *Biosensors 2022, Vol. 12, Page 167* 12, 167 (2022).

10. Song, Z., Wang, B., Zhang, Z., Yu, Y. & Lin, D. A Highly Flexible Piezoelectric Ultrasonic Sensor for Wearable Bone Density Testing. *Micromachines (Basel)* 14, (2023).

11. Mansouri, S., Alhadidi, T. & Azouz, M. Ben. Breast cancer detection using low-frequency bioimpedance device. *Breast Cancer: Targets and Therapy* 12, 109–116 (2020).

12. Rahman, A., Islam, M. T., Singh, M. J., Kibria, S. & Akhtaruzzaman, M. Electromagnetic Performances Analysis of an Ultra-wideband and Flexible Material Antenna in Microwave Breast Imaging: To Implement A Wearable Medical Bra. *Scientific Reports 2016 6:1* 6, 1–11 (2016).

13. Du, W. *et al.* Conformable ultrasound breast patch for deep tissue scanning and imaging. *Sci Adv* 9, (2023).

14. Shin, K., Leung, K., Han, F. & Jiao, J. Thermal and moisture control performance of different mastectomy bras and external breast prostheses. *Textile Research Journal* 90, 824–837 (2020).

15. Mukai, Y. & Suh, M. Development of a conformal woven fabric antenna for wearable breast hyperthermia. *Fashion and Textiles* 8, 1–12 (2021).

16. Vijayakumari, P. *et al.* Wearable transceiver with composite test-beds for breast cancer diagnosis. *Mater Today Proc* 45, 3120–3123 (2021).

17. Elsheakh, D. N., Elgendy, Y. K., Elsayed, M. E. & Eldamak, A. R. Circularly Polarized Textile Sensors for Microwave-Based Smart Bra Monitoring System. *Micromachines (Basel)* 14, (2023).

18. Won, H. J. *et al.* Diselenide-Bridged Carbon-Dot-Mediated Self-Healing, Conductive, and Adhesive Wireless Hydrogel Sensors for Label-Free Breast Cancer Detection. *ACS Nano* 14, 8409–8420 (2020).

19. Siboro, P. Y. *et al.* Harnessing HfO2 Nanoparticles for Wearable Tumor Monitoring and Sonodynamic Therapy in Advancing Cancer Care. *ACS Nano* 18, 2485–2499 (2024).

20. Ouyang, J. *et al.* Nanoaggregate Probe for Breast Cancer Metastasis through Multispectral Optoacoustic Tomography and Aggregation-Induced NIR-I/II Fluorescence Imaging. *Angewandte Chemie* 132, 10197–10207 (2020).

21. Ortega, F. G. *et al.* Sandwich-Type Electrochemical Paper-Based Immunosensor for Claudin 7 and CD81 Dual Determination on Extracellular Vesicles from Breast Cancer Patients. *Anal Chem* 93, 1143–1153 (2021).

22. Ji, D. D., Wu, M. X. & Ding, S. N. Photonic crystal barcodes assembled from dendritic silica nanoparticles for the multiplex immunoassays of ovarian cancer biomarkers. *Analytical Methods* 14, 298–305 (2022).

23. Barra, M. *et al.* Single-tube four-target lateral flow assay detects human papillomavirus types associated with majority of cervical cancers. *Anal Biochem* 688, (2024).

24. Zhang, B. *et al.* Fluorescence quenching-based signal amplification on immunochromatography test strips for dual-mode sensing of two biomarkers of breast cancer. *Nanoscale* 9, 18711–18722 (2017).

25. Su, X. *et al.* Absolute Quantification of Serum Exosomes in Patients with an SERS-Lateral Flow Strip Biosensor for Noninvasive Clinical Cancer Diagnosis. *ACS Appl Mater Interfaces* 15, 37130–37142 (2023).

26. CELLSEARCH® | mBC Clinical Trials & Case Studies. https://www.cellsearchctc.com/clinical-applications/mbc-clinical-trials-case-studies.
27. OSOM® BVBLUE® Test - Sekisui Diagnostics. https://sekisuidiagnostics.com/product/osom-bvblue-test/.
28. Vagisil Screening Kit. https://www.uspharmacist.com/article/vagisil-screening-kit.
29. CVS Health Feminine Screening Kit for Vaginal Health, 2 CT. https://www.cvs.com/shop/cvs-health-feminine-screening-kit-for-vaginal-health-2-ct-prodid-917719.
30. AZO Vaginal pH Test | Test Your Vaginal pH at Home - AZO. https://azoproducts.com/products/azo-vaginal-ph-test.
31. Vaginal infections home screening test | VagiSense®. https://www.vagisense.ca/en.
32. Cyrcadia Health | Early Detection Technology for Breast Cancer. https://cyrcadiahealth.com/.
33. iBreastExam | Better Breast Health - For All. https://www.ibreastexam.com/.
34. GE HealthCare (United States). Invenia Abus Breast Imaging Ultrasound. https://www.gehealthcare.com/products/ultrasound/breast-ultrasound/invenia-abus.
35. Sports Bra Black - SensoriaFitness. https://store.sensoriafitness.com/sports-bra-black/.
36. IcosaMed's Smart Bra for Breast Cancer Monitoring Homepage. https://icosamed.com/.
37. Wearable Device for Breast Self-Examination: Palpreast - UBORA. https://platform.ubora-biomedical.org/projects/eb95b284-014c-4709-90f4-87a1f07968b9.
38. About Ava: Women's Health Bracelet - AvaWomen. https://www.avawomen.com/ca_en/about.
39. OvuFirst Wearable Fertility Tracker. https://www.hellovio.com/en_ca/about-ovufirst/.
40. OvulaRing cycle tracker - determines your ovulation precisely. https://ovularing.com/en/.
41. kegg® fertility monitor & kegel ball | Cervical Fluid Monitoring. https://kegg.tech/.
42. Menstrual Cycle Tracker | Basal Temp Charting Tool. https://www.tempdrop.com/.
43. Oura Ring. Smart Ring for Fitness, Stress, Sleep & Health. https://ouraring.com/.
44. E4 wristband | Real-time physiological signals | Wearable PPG, EDA, Temperature, Motion sensors. https://www.empatica.com/research/e4/.
45. femSense ovulation tracker Fertility | Cycle tracker. https://www.femsense.com/en/.

46. Nuvo Solutions - FDA-cleared remote fetal monitoring. https://www.nuvocares.com/solutions.

47. Bloomlifeconnect. https://www.bloom-life.com/#Services.

48. Expertos en IoT e Inteligencia Artificial | Desarrollo de soluciones Smart. https://secmotic.com/.

49. wGT3X-BT | ActiGraph Wearable Devices. https://theactigraph.com/actigraph-wgt3x-bt.

50. DynaPort | MoveMonitor MoveTest | Monitoring device for activity and performance. https://www.mcroberts.nl/products/.

51. Osteoboost - Bone Health Technologies. https://www.bonehealthtech.com/#research-1.

52. Zaky, H. *et al.* Machine learning based model for the early detection of Gestational Diabetes Mellitus. *BMC Med Inform Decis Mak* 25, 130 (2025).

53. Zhang, Z. *et al.* Machine Learning Prediction Models for Gestational Diabetes Mellitus: Meta-analysis. *J Med Internet Res* 24, e26634 (2022).

54. Zheng, T. *et al.* A simple model to predict risk of gestational diabetes mellitus from 8 to 20 weeks of gestation in Chinese women. *BMC Pregnancy Childbirth* 19, 252 (2019).

55. Marić, I. *et al.* Early prediction of preeclampsia via machine learning. *Am J Obstet Gynecol MFM* 2, 100100 (2020).

56. Jhee, J. H. *et al.* Prediction model development of late-onset preeclampsia using machine learning-based methods. *PLoS One* 14, e0221202 (2019).

57. Mello, G. *et al.* Prediction of the Development of Pregnancy-Induced Hypertensive Disorders in High-Risk Pregnant Women by Artificial Neural Networks. *Clin Chem Lab Med* 39, (2001).

58. Khatibi, T., Hanifi, E., Sepehri, M. M. & Allahqoli, L. Proposing a machine-learning based method to predict stillbirth before and during delivery and ranking the features: nationwide retrospective cross-sectional study. *BMC Pregnancy Childbirth* 21, 202 (2021).

59. Malacova, E. *et al.* Stillbirth risk prediction using machine learning for a large cohort of births from Western Australia, 1980–2015. *Sci Rep* 10, 5354 (2020).

60. Wang, Z. *et al.* AI-assisted diagnosis of vulvovaginal candidiasis using cascaded neural networks. *Microbiol Spectr* 13, (2025).
